# Dose-sparing effects of novel adjuvants and aluminum hydroxide on two different vaccines in a neonatal mouse model

**DOI:** 10.3389/fimmu.2025.1646677

**Published:** 2025-07-31

**Authors:** Jenny Lorena Molina Estupiñan, Poorya Foroutan Pajoohian, Gabriel Kristian Pedersen, Dennis Christensen, Serena Marchi, Emanuele Montomoli, Stefanía P. Bjarnarson, Ingileif Jonsdottir, Audur Anna Aradottir Pind

**Affiliations:** ^1^ Faculty of Medicine, School of Health Sciences, University of Iceland, Reykjavík, Iceland; ^2^ Department of Immunology, Landspitali, the National University Hospital of Iceland, Reykjavík, Iceland; ^3^ Center for Vaccine Research, Statens Serum Institut, Copenhagen, Denmark; ^4^ Department of Molecular and Developmental Medicine, University of Siena, Siena, Italy; ^5^ VisMederi, Siena, Italy

**Keywords:** immunization, neonates, adjuvants, dose-sparing, antibodies, antibody-secreting cells

## Abstract

Childhood vaccination provides protection against infectious diseases, but multiple vaccinations are required to achieve this. In situations like influenza epidemics or COVID-19 pandemic, vaccine demands may exceed production capacity, highlighting the need for dose-sparing strategies. Adjuvants can boost and modulate immune responses to vaccines and could reduce the antigen doses needed to confer protection. Herein we evaluated the dose-sparing effects of the novel adjuvants dmLT, mmCT, CAF01, and CAF08b and alum (aluminum hydroxide) on primary neonatal antibody (Ab) response to a conjugate vaccine against *Streptococcus pneumoniae*, Pn1-CRM_197_, and a recombinant influenza hemagglutinin (HA) protein vaccine. The primary Ab levels of neonatal mice immunized once with a full dose of Pn1-CRM_197_ or HA were low. mmCT and CAF08b enhanced Pn1-specific IgG Abs elicited by fractional doses of Pn1-CRM_197_, providing eightfold dose sparing of the vaccine, whereas dmLT and CAF01 provided fivefold and twofold dose sparing, respectively. These adjuvants elicited protective Pn1-specific Ab levels against bacteremia (91%–63%) and pneumonia (50%–38%) in neonatal mice when combined with a half-dose of Pn1-CRM_197_. In addition, mmCT, CAF01, and CAF08b enhanced the persistence of Pn1-specific IgG Ab-secreting cells (ASCs) in bone marrow compared with a full dose of vaccine only. With the influenza HA vaccine, CAF08b provided 40-fold dose sparing, while CAF01 and mmCT provided twofold dose sparing. CAF08b induced the micro-neutralization (MN) titers above protective levels in 100% and 86% of mice receiving 1/8 and 1/40 of HA dose, respectively, and CAF01 in 88% and 50% of mice receiving 1/4 and 1/8 dose of HA, respectively, whereas only 38% of mice receiving a full-dose HA without adjuvant reached the protective MN levels. Furthermore, these adjuvants provided cross-protective Abs and ASCs against a closely related heterologous influenza strain. In contrast, aluminum hydroxide did not provide any dose-sparing effects. Collectively, our results demonstrate that mmCT, CAF01, and CAF08b enhanced the protective humoral responses and had large dose-sparing effects on both Pn1-CRM_197_ and HA vaccines, although the adjuvant effect was clearly vaccine-dependent. The results support the potential use of safe adjuvants in situations when vaccine production capacity is limited, including vaccination of pediatric populations that may be of high risk.

## 1 Introduction

Childhood vaccination against infectious diseases have a major impact on human health, preventing each year 3.5–5 million deaths from infectious diseases, including diphtheria, tetanus, pertussis, influenza, and measles (1). However, infectious diseases remain as one of the main causes of death for children under 5 years of age (2), many of which are vaccine preventable. The ideal vaccine would be a single dose given at birth at a low cost, with good safety profile, with few side effects, and that provides long-lasting protection (3). Even though protective vaccines against many diseases are available, antibody (Ab) immunity wanes 6–9 months after several primary doses in infancy, making boosters during the second year of life essential to maintain the protection and immunological memory (4). Multiple vaccinations require more doses of vaccine being manufactured, which is expensive and can be problematic in cases such as influenza or COVID-19 pandemic where the vaccine demands may exceed the vaccine production capacity. Possible solutions include dose-sparing strategies, which aim to increase vaccine immunogenicity and reduce the dose of vaccine required to confer effective immunity, often by including adjuvants into the vaccine formulation. Adjuvants can increase the specific immune response to an antigen due to their capacity to enhance both the magnitude and duration of immune responses, and some can modulate the nature of responses (5, 6). In recent years, numerous studies have demonstrated the dose-sparing capacity of several adjuvants in both animal models (7–10) and clinical trials (11–13), highlighting their use as an effective dose-sparing tool with vaccines in urgent situations.

In this study, we evaluated the dose-sparing effects of two types of adjuvants, the enterotoxin-derived adjuvants mmCT and dmLT and the cationic liposome adjuvants CAF01 and CAF08b, using a neonatal murine model (**Supplementary Table S1**). Enterotoxin-derived adjuvants, including LT-K63, mmCT, and dmLT, have been shown to overcome key limitations of the neonatal immune system (14–18). The non-toxic mmCT and dmLT were designed for mucosal application, but we have demonstrated their capabilities to enhance the induction and persistence of neonatal immune responses to pneumococcal conjugate vaccines and tetanus toxoid after both mucosal and systemic application (14, 16, 19), rendering them promising adjuvants for early-life vaccination. CAF01 is an adjuvant composed of cationic liposomes DDA (dimethyldioctadecylammonium) with the synthetic immunomodulator and mincle agonist TDB (trehalose 6,6′-dibehenate) (20) and has been shown to increase immune responses to different vaccines in adult mice (9, 21, 22) as well as neonatal mice where it was shown to induce *bona fide* germinal centers and promote long-lasting antibody responses sufficient to protect against influenza challenge following a single HA/CAF01 immunization (23, 24), making it a potent novel adjuvant. CAF08b is also a combined synergistic adjuvant composed of DDA, a TLR7/8 agonist (3M-052) along with TDB, and has been shown to activate neonatal monocyte-derived dendritic cells and enhance Th1 polarization (25), providing protective immunity against RSV in neonatal mice (26). For comparison, we used alum (aluminum hydroxide), the most widely used adjuvant for over 80 years and licensed in many pediatric vaccines, such as diphtheria, tetanus, and pneumococcal conjugate vaccines (27, 28).

To evaluate the dose-sparing effects of these adjuvants, we selected two vaccines against respiratory pathogens, highly relevant for early-life vaccination: first, a conjugate vaccine, Pn1-CRM_197_, against the extracellular bacteria *Streptococcus pneumoniae*, a significant pathogen responsible for severe and potentially life-threatening infections in young children (29, 30), and secondly, we chose a recombinant hemagglutinin (HA) protein vaccine against *influenza virus*, as it is recommended by WHO and the American Academy of Pediatrics that all children over 6 months of age are vaccinated against influenza (31, 32). Influenza pandemics and seasonal outbreaks are also clear examples of the need for dose-sparing strategies since vaccine demand often exceeds the vaccine production capacity (33). Furthermore, vaccine efficacy in neonates, especially to subunit vaccines, is suboptimal, and it has proven difficult to generate vaccine-specific immune responses at an early age, which can be improved with the use of adjuvants (23).

In this paper, we demonstrate that the adjuvants mmCT, dmLT, CAF01, and CAF08b can enhance primary humoral immune responses induced by fractional doses of the vaccine Pn1-CRM_197_, achieving protective Ab levels in neonatal mice. Thus, mmCT and CAF08b enabled dose sparing of Pn1-CRM_197_ by at least eightfold, whereas dmLT and CAF01 enabled five- and twofold dose sparing, respectively. Moreover, CAF08b enabled 40-fold dose sparing with the HA vaccine, whereas CAF01 and mmCT provided twofold dose sparing. In contrast, aluminum hydroxide did not induce dose sparing of either vaccine tested. CAF08b and CAF01 also increased the cross-protective HA antibodies to a heterologous influenza strain with reduced HA doses. Our results demonstrate that the adjuvants mmCT, dmLT, CAF01, and CAF08b have dose-sparing capacities and can potentially be used to reduce vaccine antigen dose in early-life vaccination in situations where vaccine production capacity is limited.

## 2 Materials and methods

### 2.1 Mice

NMRI mice (5 to 6 weeks old) were obtained from Taconic (Skensved, Denmark). At 1 week after arrival, we started the breeding, putting together two female mice and one male mouse for 2 weeks, then separating the females, one in each cage, and checking the cages daily for pups. At 4 weeks of age, the mice were weaned from their mothers. The animals were kept under standardized conditions at ArcticLAS vivarium facility (Reykjavík, Iceland), with free access to commercial food pellets and water and regulated temperature, daylight, and humidity. The Experimental Animal Committee of Iceland approved the protocol according to regulations 279/2002 (licence no. 2021-01-04, valid to 31.12.2024).

### 2.2 Vaccine, adjuvants, and immunizations

The Serum Institute of India (India) provided the pneumococcal conjugate vaccine Pn1-CRM_197_ which consists of a pneumococcal polysaccharide of serotype 1 (Pn1) conjugated to a non-toxic mutant of diphtheria toxoid (CRM_197_) (34). The influenza vaccine, a recombinant hemagglutinin (rHA) protein from A/Michigan/45/2015 (H1N1) influenza virus, was provided by Sanofi (MA, USA). The rHA is a full-length glycosylated protein produced in Sf9 cell line, a clonal isolate of *Spodoptera frugiperda* using baculovirus expression vector system. The adjuvants (**Supplementary Table S1**) mmCT and dmLT were manufactured as previously described (35, 36) and provided by the Vaccine Research Institute (University of Gothenburg, Sweden). Lyophilized dmLT and mmCT were resuspended in sterile nuclease-free water (Qiagen, Venlo, Netherlands) to a final concentration of 1.4 µg/mL. CAF01 and CAF08b were provided by Statens Serum Institut (Copenhagen, Denmark) and prepared as described (37). Aluminum hydroxide (alhydrogel) was purchased from Brenntag Biosector A/S (Ballerup, Denmark).

The neonatal mice (7 days old) were immunized with 50 µL of vaccine solution at the base of the tail subcutaneously (s.c.) once with a full dose of either vaccine (4 µg) or fractional doses of the vaccine (2, 1, 0.75, 0.5, and 0.1 µg) with the selected adjuvant: mmCT (5 µg/mouse), dmLT (5 µg/mouse), CAF01 (250 µg DDA/50 μg TDB), CAF08b (125 µg DDA/25 µg TDB/1 µg 3M-052), or alhydrogel (0.48% aluminum hydroxide per 1 µg of protein/mouse). The vaccine formulations were mixed 1 h prior to vaccination by mixing the vaccine solution with the adjuvant solution in sterile saline. For CAF-adjuvanted vaccine formulations (CAF01/CAF08b), vaccine solutions were mixed with TRIS (trisaminomethane) buffer provided by Statens Serum Institute at room temperature to a total volume of 50 µL and vortexed for 1 to 2 min prior to adding CAF adjuvant solution at room temperature to the solution, 100 µL at a time and vortexing for 1 to 2 min in between.

### 2.3 Blood sampling

The mice were bled bi-weekly, from weeks 2 to 8 post-vaccination, from the tail vein. The serum was isolated for measurement of anti-Pn1 or anti-HA IgG antibodies, micro-neutralization (MN), and hemagglutination inhibition assay (HAI), by centrifugation at 2,400 rpm for 10 min at room temperature and stored at -20°C.

### 2.4 Pn1- and HA-specific IgG enzyme-linked immunosorbent assay

IgG Pn1- and HA-specific antibodies were measured by enzyme-linked immunosorbent assay (ELISA) as previously described (38).

For Pn1-specific IgG antibodies, microtiter plates (MaxiSorp; Nunc AS) were coated with 100 µL of 5 μg/mL Pn1 (161-X, American Type Culture Collection, Rockville, MD, USA) in phosphate-buffered saline (PBS) solution for 5 h at 37°C (coated plates could be stored at 4°C for up to 3 weeks). The plates were then washed four times with PBS containing 0.05% Tween 20 (PBS-Tween; Sigma, Saint Louis, MO, USA) and blocked with PBS-Tween with 1% bovine serum albumin (BSA; Millipore Corporation, Bedford, MA, USA) for 1 h at room temperature in 100 µL/well. The serum samples and standard were neutralized with 500 μg/mL cell wall polysaccharide (CWPS; Statens Serum Institute, Copenhagen, Denmark) for 30 min at room temperature in a dilution ratio of 1:50. After discarding the blocking solution from plates without washing, pre-diluted serum/standard samples (standard: 1/3,000, samples dilution range: 1/50–1/500) were serially diluted in threefold dilution and incubated in duplicates for 2 h at room temperature in 100 µL/well.

For HA-specific IgG antibodies, the microtiter plates were incubated overnight at 4°C with 2.0 µg/mL HA (A/Michigan/45/2015 or A/Wisconsin/588/2019 strain (for cross-reactive Abs), Sino Biological, Beijing, China) in 0.05 M carbonate-bicarbonate buffer (pH 9.6) containing NA_2_CO_3_ (15 mM) and NaHCO_3_ (35 mM) with NaN_3_. The plates were then washed four times with PBS containing 0.05% Tween 20 (PBS-Tween; Sigma, Saint Louis, MO, USA) and blocked with PBS-Tween with 1% bovine serum albumin (BSA; Millipore Corporation, Bedford, MA, USA) for 1 h at room temperature in 100 µL/well. After discarding the blocking solution from plates without washing, pre-diluted serum/standard samples (standard: 1/4,000, serum samples dilution range 1/50–1/3,000) were serially diluted in threefold dilution and incubated in duplicates for 2 h at room temperature in 100 µL/well.

For the detection of both Pn1- and HA-specific IgG antibodies, the plates were washed (as previously described) and incubated with 1:5,000 horseradish peroxidase (HRP)-conjugated goat anti-mouse IgG (cat. no.: 1012-05, Southern Biotechnology Associates Inc., Birmingham, AL, USA) in PBS-Tween for 2 h at room temperature in 100 µL/well. Following five washes of the plates, 100 µL/well of 3,3′,5,5′-tetramethylbenzidine substrate (Kirkegaard & Perry Laboratories, Gaithersburg, MD, USA) was added for the development of the HRP enzyme reaction, and 13–15 min later, the reaction was stopped with 100 µL 0.18 M H_2_SO_4_. Absorbance was measured at 450 nm using a Multiskan FC Microplate Photometer (Thermo Scientific, Waltham, MA, USA), and optical density ranging from 0.1 to 2.0 was considered valid. Standard curves made by serial dilutions of reference serum pools from adult mice hyperimmunized with the Pn1-CRM_197_ or influenza HA vaccine were used to quantify vaccine-specific IgG Abs. The results were presented as mean log of ELISA units (EU/mL) ± standard deviation (SD). Readouts with coefficient of variation (CV) >20 were excluded, and the measurements of those samples were repeated. Representative standard curves are displayed in **Supplementary Figure S1**.

### 2.5 Micro-neutralization assay

The micro-neutralization (MN) assay was modified from a previously described procedure (40). This method is based on the capability of live virus to infect and replicate in cells. The presence of neutralizing antibodies contained in the serum of vaccine subjects is assessed by performing an ELISA assay which is based on the detection of the viral nucleoprotein (NP) expressed by the virus-infected cells forming the monolayer. Influenza live virus A/Michigan/45/2015 was propagated in Madin–Darby canine kidney (MDCK) cells by VisMederi and used in the assay at the concentration of 200 TCID_50_/100 μL [50% tissue culture infective dose (TCID50)]. Positive and negative control sera, as well as a back-titration plate for virus titration check, were included in each run. The hyperimmune serum used was specific for the strain tested, while depleted serum (serum minus IgA/IgM/IgG, S5393, Sigma-Aldrich, St. Louis, MO, USA) was used as the negative control.

The serum samples were heat-inactivated at 56°C for 1 h. Each heat-inactivated serum was twofold diluted in microtiter plates, starting from 1:10, and incubated with an equal volume of virus solution (200TCID_50_/100 μL) for 1 h at 37°C and 5% CO_2_. Then, 100 µL of MDCK cell suspension was added to the virus–sera mixture at the concentration of 1.5 × 10^5^ cells/mL; then, the plates were incubated at 37°C and 5% CO_2_ for 16–20 h. After overnight incubation, the cells were fixed, and the presence of influenza A virus NP in infected cells was detected by ELISA using the Pan Influenza A Nucleoprotein Rabbit mAb (Sino Biological) as primary Ab and HRP-labeled goat anti-rabbit IgG-Fc (Sino Biological) as secondary Ab. TMB was used as substrate for HRP, and the optical density (OD) (measured at 450 nm) of each well was determined by using ELISA reader. The OD value *x* which represents the cutoff for virus neutralization was calculated as follows:

*x* = [(average OD of VC wells) – (average OD of CC wells)]/2 + (average OD of CC wells)

where the cell control (CC) consisted of non-infected cells and the virus control (VC) consisted of infected cells without addition of serum. All wells with an OD (450 nm) below or equal to “*x*” were considered positive for neutralization activity. The neutralizing titer was determined as the reciprocal of the highest serum dilution with an OD ≤*x*. Indicative seroprotection rate was defined as the percentage of vaccine recipients with serum MN titer ≥20 after vaccination.

### 2.6 Hemagglutination inhibition assay

The hemagglutination inhibition assay (HAI) measurement was carried out by following the VisMederi procedures. The influenza antigen strain A/Wisconsin/588/2019, heterologous H1N1 closely related to the vaccine strain (A/Michigan/45/2015), was propagated in MDCK cells. Viral antigen was diluted at the standard concentration of 160 hemagglutinating units (HAU)/mL, and the correctness of antigen dilution was checked out through back-titration in every run. Positive and negative control sera were included in each run. The hyperimmune serum used as the positive control was specific for the strain tested, while depleted serum (serum minus IgA/IgM/IgG, S5393, Sigma-Aldrich) was used as the negative control.

The serum samples were treated with a receptor-destroying enzyme (RDE, Denka Seiken, Tokyo, Japan) in a ratio of 1:3. The sera were incubated overnight with RDE solution and then heat-inactivated at 56°C for 1 h. After RDE treatment, adsorption with turkey red blood cells (RBCs) was carried out to remove non-specific agglutinins. A turkey RBC solution at 12.5% in Dulbecco PBS (Thermo Scientific) was added to the serum–RDE mixture in a ratio of 1:1, getting a final serum dilution of 1:10.

Twofold serial dilutions starting from 1:10 were performed for each serum in duplicate in “V”-bottomed 96-well plates. The antigen solution (4 HAU/25 μL) was added to each serum dilution, and the plates were incubated for 1 h at room temperature. A 0.5% solution of turkey RBCs was added to each well, and the plates were incubated for 1 h at room temperature. The HA protein is able to agglutinate RBCs due to its binding affinity to the surface glycoprotein of erythrocytes, and antibodies may interfere with this binding recognizing the virus antigen. This phenomenon produces an inhibition of hemagglutination, resulting in a change in the appearance of the well (41, 42). The readout was performed by naked eye, distinguishing between the presence of hemagglutination and inhibition of it. The HAI titer was calculated as the reciprocal value of the highest serum dilution in which the hemagglutination was still inhibited. Seroprotection rate was defined as the percentage of vaccine recipients with serum HAI titer ≥40 after vaccination. If the initial dilution did not give a positive titer, the titer was recorded as half of the minimum detectable titer for calculation purposes (e.g., 5).

### 2.7 Isolation of mononuclear cells from spleen and bone marrow

Isolation of mononuclear cells was performed as previously described (43). Spleen, femur, and tibia were dissected from mice at 8 weeks post-immunization. The bone marrow (BM) was flushed out of the bones by injection of 2.0 mL of RPMI 1640 medium (RPMI-1640; Gibco, Fisher Scientific, Paisley, UK) containing 25 mM hepes buffer (Gibco), 2 mM L-glutamine (Gibco), 100 U/mL penicillin/100 µg/mL streptomycin (Gibco), and 10% fetal calf serum (FCS; Gibco). The spleens were pressed through a sterile 100-µm nylon cell strainer (BD Biosciences, San Jose, CA, USA) to obtain single cell suspensions which were maintained in sterile Hanks balanced salt solution (HBSS, Gibco) with 100 U/mL penicillin/100 µg/mL streptomycin and 2% FCS. The spleen and BM single-cell suspensions were incubated with ammonium chloride buffer (pH 7.2) for 5 min to lyse red blood cells. After washing, the cells were resuspended in 10 mL of tissue culture media and kept on ice for 10 min to allow tissue fragments to settle in the bottom of the tubes. Then, the top 9.0 mL was gently moved to a new tube and centrifuged at 1,000 rpm for 10 min at 4°C. The number of cells in each tube was calculated by counting the cells stained with trypan blue (Sigma) in C-CHIP disposable hematocytometers (NanoEntek, Seoul, Korea) using a microscope (Leica, Biosystems, Wetzlar, Germany). Each cell suspension was adjusted to 10^8^ cells per milliliter to perform the ELISPOT analysis.

### 2.8 Pn1- and HA-specific enzyme-linked immunospot

Pn1- and HA-specific IgG^+^ ASCs were enumerated by enzyme-linked immunospot (ELISpot) in bone marrow and spleen 8 weeks after immunization as previously described (44). MultiScreen-IP 96-well sterile plates (Millipore Corporation, Bedford, MA, USA) were coated with 20 µg/mL Pn1 (ATCC) or 4 µg/mL HA (A/Wisconsin/588/2019 strain, Sino Biological) and incubated overnight at 37°C. After washing once with PBS containing 0.05% Tween 20 (PBS-Tween; Sigma) and three times with PBS, RPMI 1640 (Thermo Fisher, UK) with 10% fetal bovine serum (Thermo Fisher), penicillin/streptavidin (Thermo Fisher), and L-glutamine (Thermo Fisher) was incubated for 1 h at 37°C for blocking. Single-cell suspension isolated from bone marrow and spleens, as described above, were incubated in duplicate wells for each of four threefold dilutions starting with 1 × 10^7^ cells in 100 µL per well for 5 h at 37°C. Then, the plates were washed and incubated with alkaline phosphatase (AP)-labeled-goat anti-mouse IgG (cat. no.: 1030-04, Southern Biotechnology Associates) at 4°C overnight. The development of the AP enzyme reaction and precipitation of the colored product around each antibody-secreting cell (ASC) was achieved by 5-bromo-4-chloro-3-indolylphosphate and nitroblue tetrazolium (NBT) in AP development buffer (Bio-Rad Labs, Hercules, CA, USA). Pn1- and HA-specific ASCs (spots) were counted using the ELISPOT reader ImmunoSpot R S6 ULTIMATE with ImmunoSpot R SOFTWARE (Cellular Technology Limited (CTL) Europe, Bonn, Germany).

The results are presented as the number of ASC per 10^6^ cells calculated as mean cell number in wells with number of spots within the recommended range (10–200) after correcting for the dilution.

### 2.9 Statistical analysis

Mann–Whitney *U*-test was used for statistical comparison between groups, and Spearman rank-order correlation was used to assess correlation using GraphPad Prism 10.2.3 (GraphPad Software, La Jolla, CA, USA). *P*-values below 0.05 were considered statistically significant (*p ≤ 0.05, **p ≤ 0.01, ***p ≤ 0.001).

## 3 Results

### 3.1 Adjuvants mmCT, dmLT, CAF01, and CAF08b enhance the protective antibody response after neonatal immunization, providing dose sparing for the vaccine Pn1-CRM_197_

We and others have shown how adjuvants can overcome limitations of the early life immune system, enhancing responsiveness to a variety of vaccine antigens in neonatal mice (14, 15, 23, 45–48). Thus, it is conceivable that by increasing vaccine immunogenicity, adjuvants could minimize the dose of antigen necessary to reach protective immunity.

**Figure 1:**
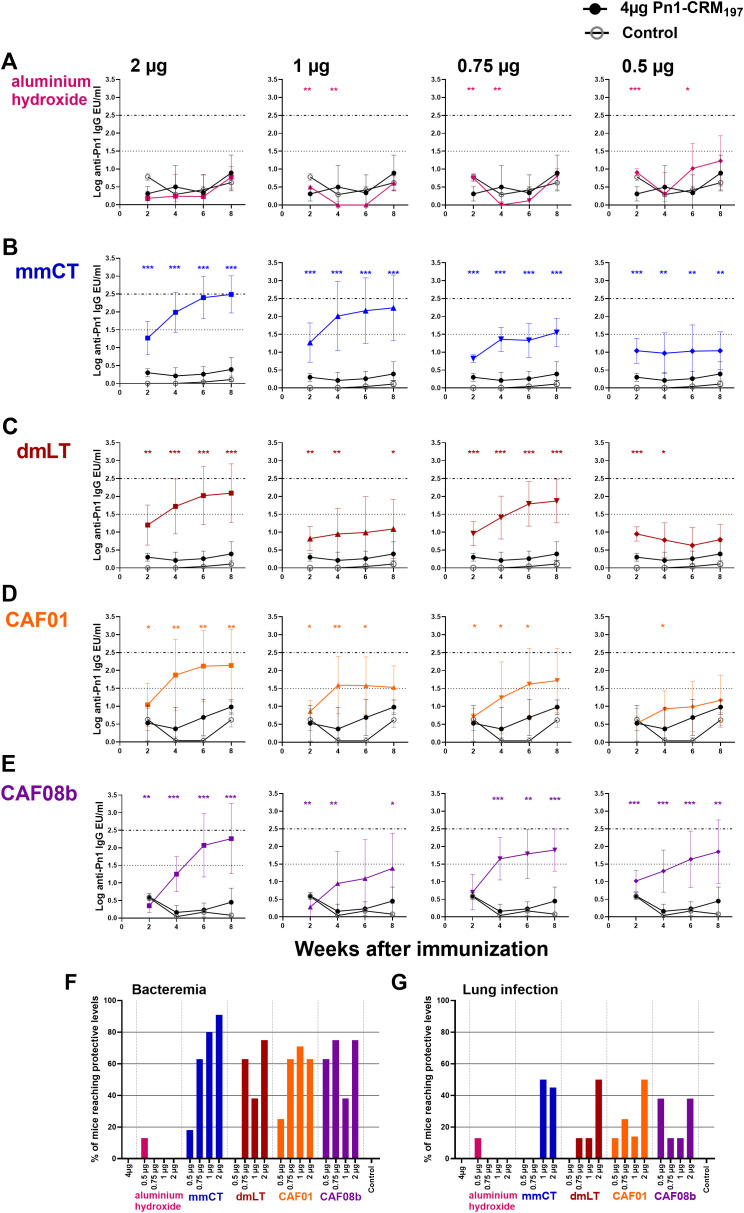
Adjuvants mmCT, dmLT, CAF01, and CAF08b enhance humoral immune responses, providing dose sparing for the vaccine Pn1-CRM_197_. Pn1-specific serum Ab levels at 2, 4, 6, and 8 weeks after s.c. immunization of neonatal mice with fractional doses (2, 1, 0.75, and 0.5 μg) of Pn1-CRM197 with adjuvants aluminum hydroxide **(A)**, mmCT **(B)**, dmLT **(C)**, CAF01 **(D)**, or CAF08b **(E)** or with a full dose (4 μg) of Pn1-CRM197 alone (black-filled circle), and an unimmunized control group (grey open circle) **(A–E)**. The results are expressed as IgG levels (log mean EU/mL ± SD) in seven to nine mice per group, and statistical difference was calculated using Mann–Whitney *U*-test where adjuvant groups were compared to the 4-µg-of-vaccine-only group. *p ≤ 0.05, **p ≤ 0.01, ***p ≤ 0.001. The dotted lines represent protective IgG Ab levels for pneumococcal bacteremia (log 1.5) and lung infection (log 2.5). Based on these values, the percentages of mice in each group that reached protective Ab levels against bacteremia **(F)** and lung infection **(G)** at 8 weeks after immunization were calculated. The figure shows the results from four independent experiments, where mmCT and dmLT were assessed in the same experiment, and the rest of the adjuvants were assessed in different experiments.

To evaluate the antigen dose-sparing capacity of the adjuvants mmCT, dmLT, CAF01, and CAF08b, neonatal mice were immunized subcutaneously once with a full dose (4 µg) of Pn1-CRM_197_ or fractional doses (2, 1, 0.75, and 0.5 µg) of Pn1-CRM_197_ with adjuvant. Sera obtained 2, 4, 6, and 8 weeks after immunization were used to measure Pn1-specific IgG Abs, and adjuvanted groups were compared to the full-dose vaccine only group. The IgG Ab levels of neonatal mice immunized with a full dose of Pn1-CRM_197_ alone were low. Inclusion of the adjuvants mmCT, dmLT, CAF01, and CAF08b in the vaccine formulation significantly enhanced the Pn1-specific IgG Ab responses elicited by fractional doses of Pn1-CRM_197_ compared with a full dose of Pn1-CRM_197_ without an adjuvant up to 8 weeks after immunization (**Figure 1**). In contrast, immunization with fractional doses of Pn1-CRM_197_ and aluminum hydroxide elicited low IgG Abs, comparable to those of Pn1-CRM_197_ alone, rendering aluminum hydroxide unable to provide a dose-sparing effect of the vaccine Pn1-CRM_197_ (**Figure 1A**). By including mmCT and CAF08b in the vaccine formulation, it was possible to reduce the vaccine dose down to 0.5 µg while still significantly enhancing Pn1-specific IgG Abs compared with 4 µg of Pn1-CRM_197_ alone, providing at least eightfold sparing of the Pn1-CRM_197_ vaccine dose (**Figures 1B, E**). By including dmLT in the vaccine formulation, the Pn1-CRM_197_ dose could likewise be lowered to 0.75 µg, indicating that dmLT can promote dose sparing by reducing the Pn1-CRM_197_ vaccine dose by at least fivefold (**Figure 1C**). Lastly, the inclusion of CAF01 enabled twofold sparing of the Pn1-CRM_197_ dose (**Figure 1D**).

Our group has established a protective Pn1-specific IgG Ab threshold in adult mice in an intranasal pneumococcal infection murine model, where IgG levels above log 1.3 EU/mL protect against bacteremia and levels above log 2.5 EU/mL protect against lung infection regardless of the immunization route, following immunization with a pneumococcal conjugate vaccine Pnc1-TT (39). These thresholds have been confirmed across various immunization settings (adult, infant, neonatal, and maternal), although neonatal mice require a slightly higher threshold for bacteremia at log 1.5 EU/mL (49–53). We have confirmed the protective thresholds of log 1.5 and 2.5 EU/mL for pneumococcal bacteremia and lung infection, respectively, in over 90% of subcutaneously immunized neonatal mice using the pneumococcal conjugate vaccine Pn1-CRM_197_ (**Supplementary Figure S2**). Based on this, we calculated the percentage of mice in each group that reached protective Ab levels against bacteremia or lung infection. Importantly, none of the mice immunized once with 4 µg of Pn1-CRM_197_ alone reached protective Ab levels against bacteremia or lung infection. However, one immunization with half dose (2 µg) and 1/5 (0.75 µg) of Pn1-CRM_197_ with mmCT, dmLT, CAF01, or CAF08b, but not aluminum hydroxide, induced protective Ab levels against bacteremia (91%–63% and 75%–63%, respectively) and pneumonia (38-50% half dose) 8 weeks after immunization, which were significantly higher than those of the full dose (4 µg) of Pn1-CRM_197_ without adjuvant (0% and 0%, respectively) (**Figures 1F, G**), demonstrating that even a fractional dose of Pn1-CRM_197_ with these adjuvants induced a superior protective vaccine-specific Ab responses than a full dose of Pn1-CRM_197_ alone.

### 3.2 mmCT and CAF08b improve the persistence of antibodies and antibody-secreting cells following neonatal immunization with fractional doses of Pn1-CRM_197_

**Figure 2:**
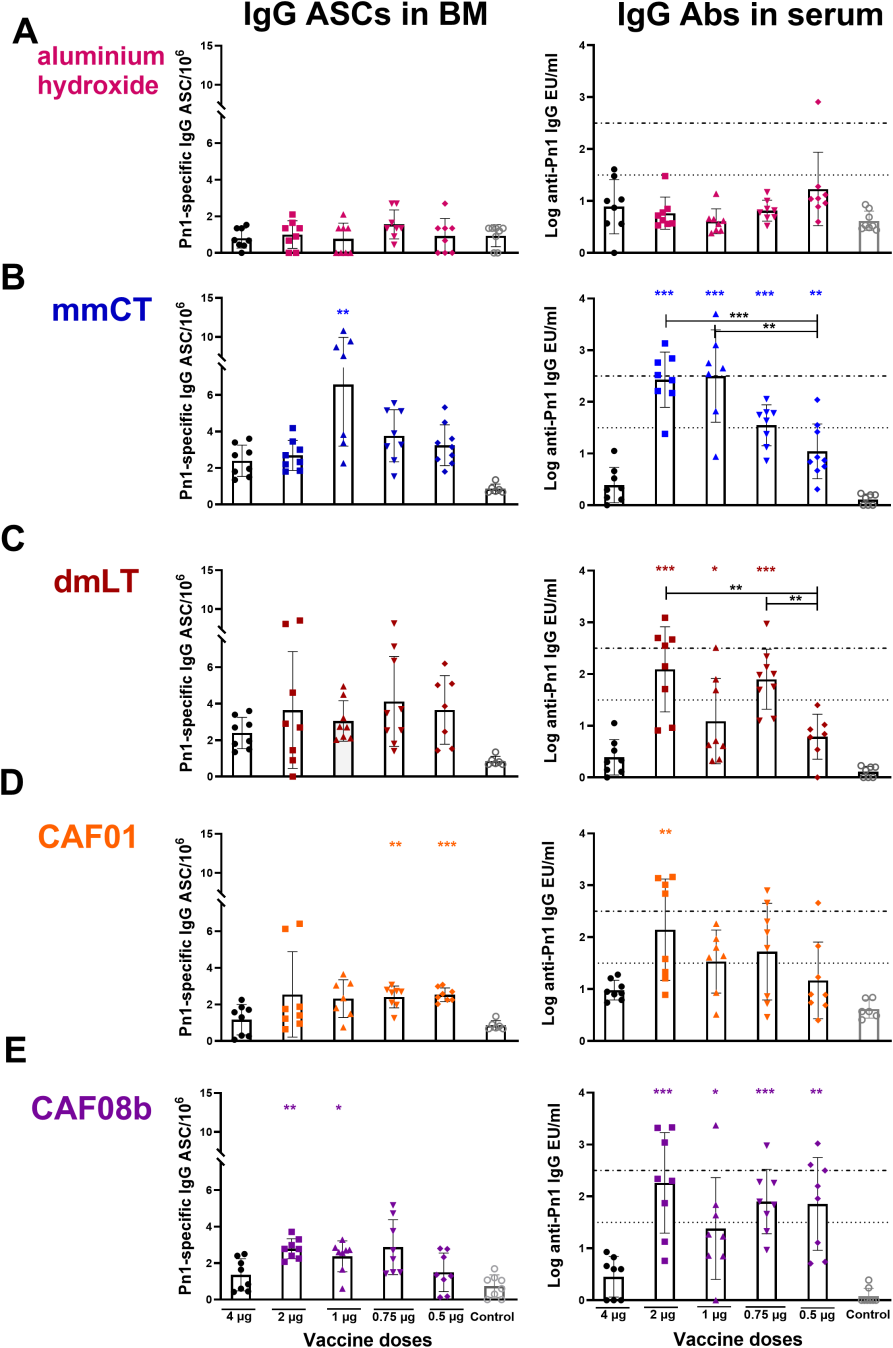
Dose-sparing effects of the adjuvants on the persistence of IgG immune response. Number of Pn1-specific IgG^+^ ASCs in BM and Pn1-specific serum Ab levels at 8 weeks after s.c. immunization of neonatal mice with fractional doses (2, 1, 0.75, and 0.5 µg) of Pn1-CRM_197_ with adjuvants aluminum hydroxide **(A)**, mmCT **(B)**, dmLT **(C)**, CAF01 **(D)**, or CAF08b **(E)** or with a full dose (4 µg) of Pn1-CRM_197_ alone (black-filled circle, **(A–E)**. The results are expressed as the number of spots/10^6^ cells (mean ± SD) and IgG levels (log mean EU/mL ± SD) in eight mice per group, and statistical difference was calculated using Mann–Whitney *U*-test where the adjuvant groups were compared to the 4-µg-of-vaccine-only group (colored stars) and fractional doses of adjuvanted groups were compared between each other (black stars). *p ≤ 0.05, **p ≤ 0.01, ***p ≤ 0.001. The results shown are from four independent experiments, where mmCT and dmLT were assessed in the same experiment, and the rest of the adjuvants were assessed in different experiments.

We have demonstrated the potential of some adjuvants to induce vaccine-specific Ab-secreting cells (ASCs) and prolong their persistence in the bone marrow (BM) following immunization of neonatal mice (14–16, 44, 55). Therefore, we wanted to assess if fractional doses of the vaccine with the adjuvants could enhance the persistence of the IgG immune response. To do so, we measured IgG^+^ ASCs in BM 8 weeks after immunization with fractional doses of the vaccine Pn1-CRM_197_ with mmCT, dmLT, CAF01, CAF08b, or aluminum hydroxide and compared them with the full dose of the vaccine alone. Immunization with fractional doses of the vaccine and aluminum hydroxide did not enhance ASCs in the BM nor IgG in serum as the levels were comparable to the vaccine-alone group (**Figure 2A**). The only fractional dose given with mmCT that had a higher number of Pn1-specific IgG^+^ ASCs in BM at this late time point compared to immunization with 4 µg of vaccine alone was 1 µg, even though all fractional doses with mmCT increased the IgG level in serum (**Figure 2B**). CAF01 increased the number of Pn1-specific IgG^+^ ASCs in BM when co-administered with 0.75 and 0.5 µg of Pn1-CRM_197_ compared with 4 µg of the vaccine alone (**Figure 2D**). However, the serum IgG Ab levels in these mice were comparable with those in the vaccine-alone group. The mice immunized with 2 and 1 µg of Pn1-CRM_197_ and CAF08b adjuvant had a higher number of Pn1-specific IgG^+^ ASCs in BM than mice the immunized with 4 µg of vaccine alone, and all fractional vaccine doses with CAF08b had higher IgG Ab levels in the serum (**Figure 2E**). Lastly, dmLT did not increase the number of Pn1-specific ASCs in BM (**Figure 2C**). However, it should be noted that even though mmCT and dmLT enhanced the Ab response compared to vaccine alone, a dose-dependent decrease in vaccine-specific IgG Abs was observed, where 0.5 µg of Pn1-CRM_197_ with mmCT induced a significantly lower level of IgG Abs than 2 and 1 µg of the vaccine with the same adjuvant (**Figure 2B**), and 0.5 µg of Pn1-CRM_197_ with dmLT induced a lower Ab response than 2 and 0.75 µg of Pn1-CRM_197_ with dmLT (**Figure 2C**). The results indicate that in our experimental setup the adjuvants mmCT and CAF08b are effective inducers of persistent Ab responses that are essential for protection from pneumococcal disease (39, 50, 54, 56). However, we and others have demonstrated that it is challenging to induce sustained polysaccharide-specific ASCs in the bone marrow after only one immunization at an early age. Therefore, reducing the vaccine dose with a booster vaccination may be more effective.

### 3.3 Dose-sparing effect of mmCT, CAF01, and CAF08b after immunization of neonatal mice with influenza vaccine HA

**Figure 3:**
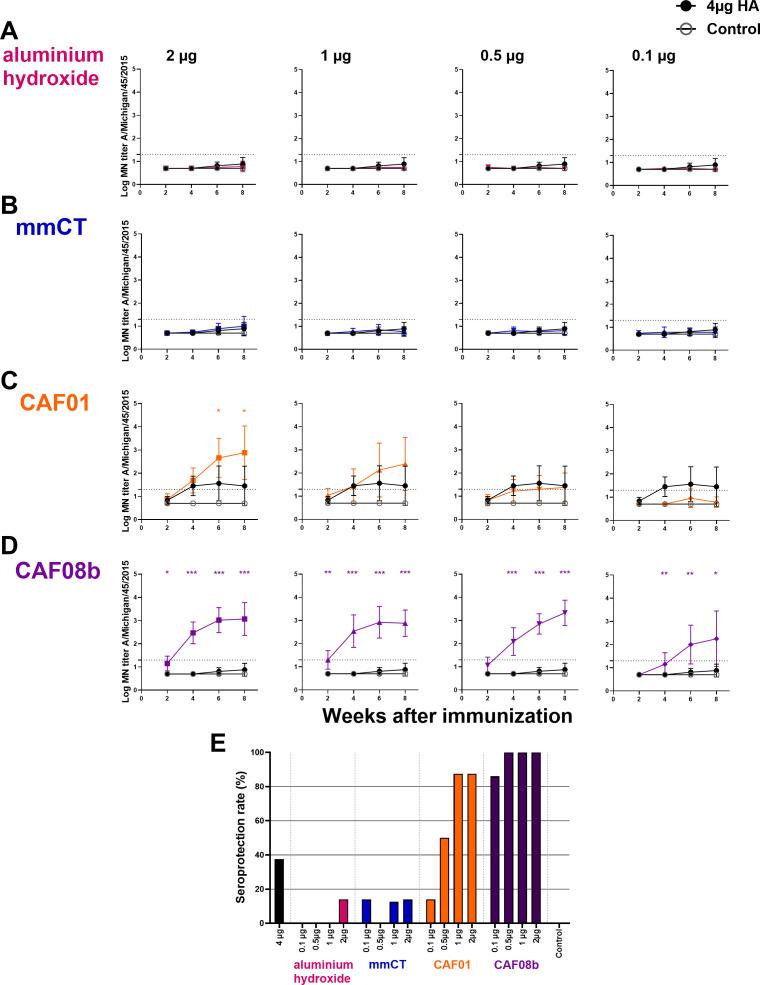
Adjuvants CAF01 and CAF08b enhance micro-neutralization (MN) titers providing seroprotection against influenza strain A/Michigan/45/2015. MN assays were performed in serum Ab levels 2, 4, 6 and 8 weeks after s.c. immunization of neonatal mice with fractional doses (2, 1, 0.5 and 0.1 µg) of HA with adjuvants aluminium hydroxide **(A)**, mmCT **(B)**, CAF01 **(C)** or CAF08b **(D)**; or with a full dose (4µg) of HA alone (black-filled circle), and an unimmunized control group (gey open circle) **(A–D)**. The results are expressed as log MN titers (mean ± SD) in eight mice per group, and statistical difference was calculated using Mann–Whitney *U*-test where adjuvant groups were compared to the 4-µg-of-vaccine-only group. *p ≤ 0.05, **p ≤ 0.01, ***p ≤ 0.001. The dotted line represents seroprotective MN titer against influenza (≥20). Seroprotection rate was defined as the percentage of mice with serum MN titer ≥20 at 8 weeks after immunization **(E)**. The results are from three independent experiments, where mmCT and aluminum hydroxide were assessed in the same experiment, and the rest of the adjuvants were assessed in different experiments.

Herein several of the adjuvants tested possess dose-sparing properties with a pneumococcal conjugate vaccine. We hypothesized that the dose-sparing effects observed with the pneumococcal conjugate vaccine might extend to other types of vaccines. Therefore, we evaluated the dose-sparing capacity of mmCT, CAF01, CAF08b, and aluminum hydroxide to a recombinant influenza HA protein vaccine. This is especially relevant, as the demand for influenza vaccines during pandemics and seasonal outbreaks often exceeds the production capacity. For this, neonatal mice were immunized subcutaneously once with full-dose HA (4 µg) or fractional doses of HA (2, 1, 0.5, and 0.1 µg) with mmCT, CAF01, CAF08b, or aluminum hydroxide. Sera obtained 2, 4, 6, and 8 weeks after immunization were used to measure protective Abs by MN assay, and the adjuvanted groups were compared to the full-dose HA-vaccine-only group. The MN assay has been reported to be more sensitive than the HAI assay for the detection of influenza virus-neutralizing antibodies (57, 58), and a MN titer of ≥20 has been suggested as an indicative correlate of protection and corresponds approximately to an HAI titer of ≥40, the conventional seroprotection threshold (58, 59). The mice immunized with a full dose of HA alone or with fractional doses of HA and aluminum hydroxide or mmCT generated low levels of neutralizing antibodies to the influenza vaccine strain (A/Michigan/45/2015) (**Figures 3A, B**). In contrast, fractional doses of HA combined with CAF01 or CAF08b elicited significantly higher MN titers, with CAF08b achieving a 40-fold (**Figure 3D**) and with CAF01 a twofold dose-sparing effect (**Figure 3C**), whereas mmCT and aluminum hydroxide showed no dose-sparing effects (**Figures 3A, B**). Importantly, CAF08b induced protective MN titers (≥20) in 100% of mice with three of the fractional doses of HA (0.5, 1, and 2 µg) and in 86% of mice receiving 0.1 µg HA dose at 8 weeks after one immunization (**Figure 3E**). The seroprotection rate was 88% in mice receiving CAF01 with 2 and 1 µg of HA and 50% in mice with 0.5 µg HA. In contrast, only 14% of mice given a half dose of HA (2 µg) with either aluminum hydroxide or mmCT achieved protective MN titers against the immunizing strain compared to 38% of mice receiving the full HA dose (4 µg) without adjuvant. We also measured IgG-specific Abs in serum 6 and 8 weeks after immunization, and the adjuvanted groups were compared to the full-dose vaccine-only group. The mice immunized with a full dose of HA alone or with fractional doses of HA and aluminum hydroxide generated low levels of HA-specific IgG (**Supplementary Figure S3A**). In contrast, the mice immunized with fractional doses of HA combined with mmCT, CAF01, or CAF08b elicited significantly higher IgG levels, where inclusion of mmCT or CAF01 with the HA vaccine enabled twofold dose sparing (**Supplementary Figures S3B, C**), but CAF08b provided 40-fold dose sparing (**Supplementary Figure S3D**). Additionally, there was a strong correlation between IgG-specific Ab levels and MN titers specific for the vaccine strain (Spearman *r* = 0.82, *p* < 0.0001, **Supplementary Figure S4**).

Taken together, our results suggest that CAF08b and CAF01 enhance the immunogenicity of the HA vaccine in neonatal mice and possess effective dose-sparing properties for the HA vaccine.

### 3.4 CAF01 and CAF08b enhance the cross-protective antibodies and ASCs following neonatal immunization with HA

**Figure 4:**
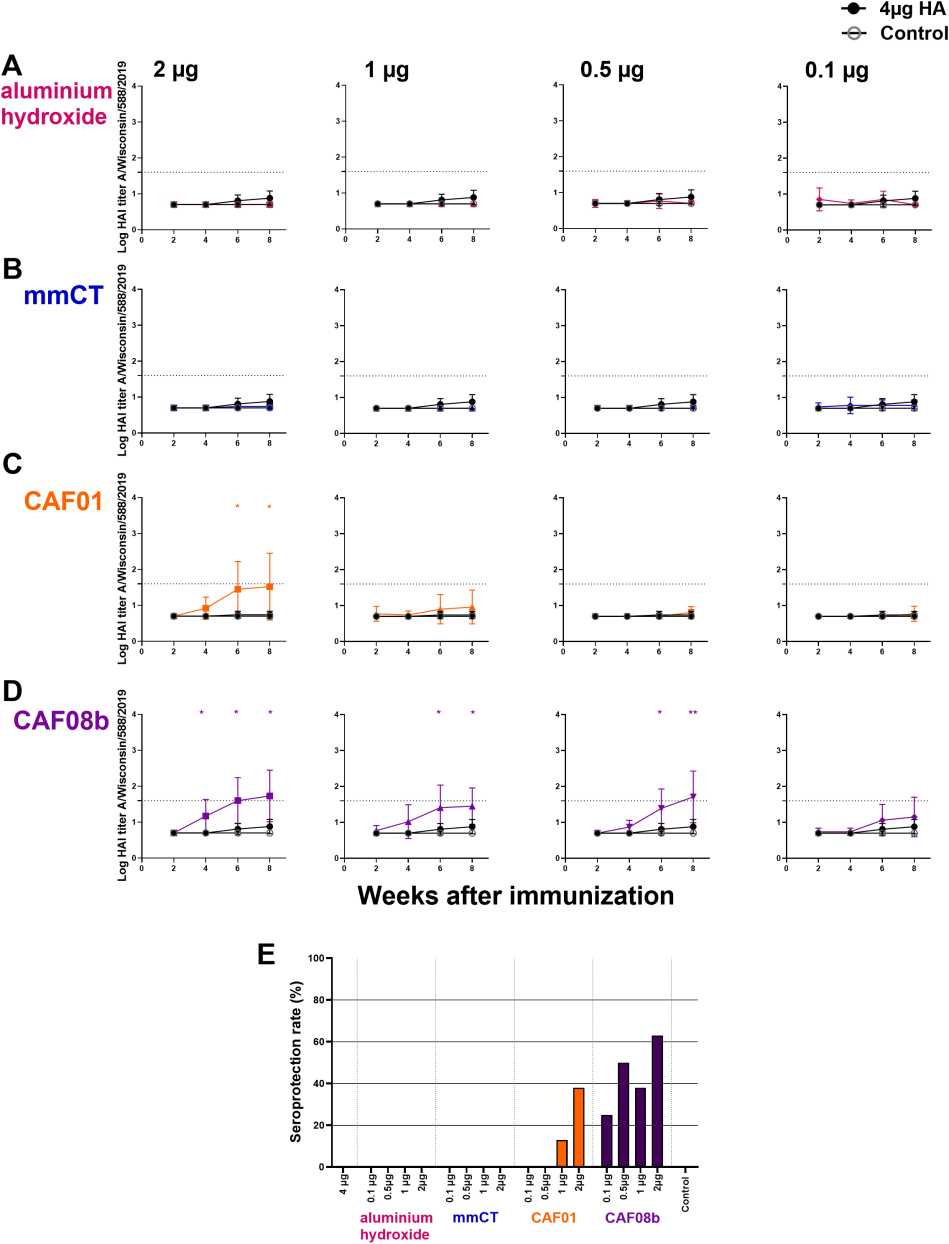
Cross-protective effects of mmCT, CAF01, and CAF08b on hemagglutination inhibition (HAI) titers. HAI Ab titers against a closely related heterologous virus strain (A/Wisconsin/588/2019) in mouse sera 2, 4, 6 and 8 weeks after s.c. immunization of neonatal mice with fractional doses (2, 1, 0.5 and 0.1 µg) of HA with adjuvants aluminium hydroxide **(A)**, mmCT **(B)**, CAF01 **(C)** or CAF08b **(D)**, or with a full dose (4µg) of HA alone (black filled circle), and an unimmunized control group (grey open circle) **(A–D)**. The results are expressed as log of geometric mean titers (mean ± SD) in eight mice per group, and statistical difference was calculated using Mann–Whitney *U*-test where adjuvant groups were compared to the 4-µg-of-vaccine-only group. *p ≤ 0.05, **p ≤ 0.01. The dotted line represents seroprotective HAI titer against influenza (≥40). Seroprotection rate was defined as the percentage of mice with serum HAI titer ≥40 at 8 weeks after immunization **(E)**. The results are from three independent experiments, where mmCT and aluminum hydroxide were assessed in the same experiment, and the rest of the adjuvants were assessed in different experiments.

To evaluate the effect of these adjuvants on cross-protective antibodies against a heterologous but closely related HA strain, we measured their protective capacity against A/Wisconsin/588/2019 by HAI assay. An HAI titer of ≥40, first described in 1972 as providing 50% protection (60), is widely used as a surrogate correlate of protection (32, 61–63). The mice immunized with a full dose of HA vaccine alone had no protective cross-reactive HAI titers at baseline, and there were no differences in the cross-reactive HAI titers between the mice immunized with a full dose of HA alone and the mice immunized with fractional doses of HA and aluminum hydroxide or mmCT (**Figures 4A, B**). However, 2 µg of HA with CAF01 and 2, 1, and 0.5 µg of HA with CAF08b had higher cross-reactive HAI titers than that induced by a full dose of vaccine alone 6 and 8 weeks after immunization (**Figures 4C, D**). Thus, eightfold and twofold reduction of the HA dose for CAF08b and CAF01, respectively, still induced enhanced cross-reactive Abs compared with the full-dose HA vaccine alone. In line with that, one immunization with 2 µg of HA with CAF08b or CAF01 induced cross-reactive seroprotection against closely related heterologous influenza strain in 63% and 38% of mice, respectively (**Figure 4E**).

Cross-reactive anti-HA IgG levels were also measured by ELISA and were low in mice receiving full-dose HA alone or with aluminum hydroxide (**Supplementary Figure S5A**). In contrast, mice immunized with fractional doses of HA combined with mmCT, CAF01, or CAF08b elicited significantly higher IgG Ab levels against the heterologous HA, where reduction of the HA dose by 40-fold with CAF08b (**Supplementary Figure S5D**) and by eightfold with mmCT or CAF01 still resulted in enhanced cross-reactive Abs (**Supplementary Figure S5B, C**). Furthermore, there was a positive strong correlation between cross-reactive IgG Ab levels and HAI titers against the heterologous strain (Spearman *r* = 0.65, *p* < 0.0001, **Supplementary Figure S6**). Significant correlations were also found between protective capacity against the vaccine strain and the closely related heterologous strain, i.e., MN titers against the vaccine strain and HAI titers against the heterologous strains (*r* = 0.64, *p* < 0.0001).

Next, we assessed if delivering lower doses of the influenza vaccine with the adjuvants mmCT, CAF01, CAF08b, or aluminum hydroxide affected the persistence of cross-reactive IgG^+^ anti-HA ASCs in BM 8 weeks after the immunization of neonatal mice. Similar to what we observed for Pn1-CRM_197_, mice immunized with fractional doses of HA and aluminum hydroxide had low levels of both cross-reactive anti-HA IgG^+^ ASCs and IgG Abs in serum and were comparable to mice immunized with a full dose of vaccine alone (**Supplementary Figure S7A**). The mice immunized with 2, 1, and 0.5 µg of HA and mmCT had a higher number of cross-reactive anti-HA IgG^+^ ASCs in BM and higher serum IgG Abs than mice immunized with 4 µg of vaccine alone (**Supplementary Figure S7B**). All fractional doses of HA with CAF01 or CAF08b elicited higher numbers of cross-reactive anti-HA IgG^+^ ASCs in BM and higher levels of IgG Abs in serum than 4 µg of vaccine alone (**Supplementary Figures S7C, D**). Furthermore, the HA vaccine with mmCT or CAF01 showed a dose-dependent decrease in cross-reactive anti-HA IgG Abs, where 0.1 µg of HA with mmCT or CAF01 induced significantly lower Ab responses than 2 µg of HA with the same adjuvant (**Supplementary Figures 7B, C**). It is worth noting that immunization with all fractional doses of HA and CAF08b or 2 and 1 µg of HA with CAF01 additionally enhanced the number of cross-reactive IgG^+^ ASCs in spleen at this late time point, 8 weeks after immunization (**Supplementary Figure S8**). These results indicate that CAF01 and CAF08b, and mmCT to a lesser degree, effectively promote a stronger and more persistent immune response even with fractional doses of the HA vaccine than HA with aluminum hydroxide or full-dose HA alone. Importantly, CAF08b and CAF01 enhanced the protective capacity to a heterologous closely related influenza strain, even when the antigen dose was reduced.

## 4 Discussion

Vaccine shortages during epidemics have highlighted a critical gap in global health preparedness, prompting the researchers to explore innovative solutions. One promising avenue is the use of adjuvants to enhance specific immune responses to vaccines. By incorporating adjuvants, vaccine formulations can improve the efficacy, potentially allowing for lower doses of the vaccine antigen (64), increasing vaccination coverage, and enabling quicker responses to outbreaks. As the world faces increasing health threats, adjuvants could be a key strategy in mitigating vaccine shortages and protecting the population more effectively (65, 66). In this study, we evaluated the dose-sparing and immunostimulatory effects of four novel adjuvants and the adjuvant aluminum hydroxide following the neonatal immunization of mice with vaccines against pneumococcus and influenza virus. We demonstrate that mmCT, CAF01, and, in particular, CAF08b enhanced the induction and persistence of humoral responses and provided large dose-sparing effects on both vaccines, even though the adjuvant effect was, to some extent, vaccine dependent.

Incorporating adjuvants into neonatal vaccine formulations could significantly improve the functionality of the immature neonatal immune system. This approach may accelerate immune maturation, improve antigen presentation, and increase T and B cell co-stimulatory signals, effectively overcoming the typically low vaccine responses in neonates (23, 24, 44, 47). Adjuvants may specifically enhance T follicular helper (Tfh) cell responses, elevate the frequency and quality of germinal center and memory B cells, and promote the production and persistence of high-affinity antibodies (23, 24). Additionally, they could strengthen cellular immunity by stimulating Th1 responses, contributing to a more robust and protective immune response (67, 68). Our group has demonstrated the ability of mmCT to enhance primary immune responses in neonatal mice, specifically increasing APRIL and BCMA expression following s.c. immunization with tetanus toxoid (TT) vaccine (16) and enhancing GC reaction, Abs, and ASCs after s.c. immunization with TT and the conjugate vaccines Pnc1-TT and Pn1-CRM_197_ (14–16, 55). mmCT also increased IgA Abs in serum and mucosa and IgA^+^ ASCs following intranasal immunization with Pn1-CRM_197_, although a higher dose of the vaccine was needed to reach comparable IgG Ab levels to s.c. immunization (14). Herein we demonstrate that mmCT enhances vaccine-specific serum IgG Abs induced by fractional doses of both Pn1-CRM_197_ and influenza HA vaccines. Using 4 µg as the full dose, mmCT achieved eightfold dose sparing of Pn1-CRM_197_ and twofold dose sparing of HA, and when the persistence of the response was evaluated, immunization with mmCT and fractional doses of Pn1-CRM_197_ induced higher number of IgG^+^ ASCs than the full dose. However, after one immunization with HA and mmCT, the mice did not induce protective Ab titers. These results emphasize the importance of using the proper adjuvant for specific vaccines since mmCT seems to be a better adjuvant for induction of protective immunity by Pn1-CRM_197_ than by the influenza HA vaccine.

We also evaluated the dose-sparing effects of another enterotoxin-based adjuvant, dmLT, with the Pn1-CRM_197_, where dmLT reached fivefold dose sparing of the vaccine. However, when evaluating the persistence of the immune response, we observed no difference in the number of Pn1-specific IgG^+^ ASCs compared to the full dose of Pn1-CRM_197_ alone, even though a trend for a higher number of Pn1-specific IgG^+^ ASCs was observed in some of the groups receiving the dmLT with fractional doses of the vaccine. Similarly, we have reported a more pronounced effect of mmCT than dmLT following neonatal immunization with Pn1-CRM_197_ (14). Nonetheless, dmLT has been shown to possess effective dose-sparing properties in adult mice where its inclusion could reduce the dose of inactivated polio vaccine while still enhancing the mucosal immunity and longevity of humoral responses (7). Furthermore, preclinical and clinical studies have confirmed the safety and effectiveness of dmLT and its ability to induce protective immunity when used with the oral inactivated enterotoxigenic *Escherichia coli* (ETEC) vaccine, both in human adults and infants (69–71). Thus, it is not clear whether dmLT is suboptimal compared to mmCT with the Pn1-CRM_197_ vaccine in our neonatal mouse model or if mmCT is better suited for neonatal immunization, but this may depend on the vaccine and the type of immune response required for protection against the pathogen.

In this study, we also assessed the dose-sparing effects of two cationic liposomes DDA-based parenteral adjuvants, CAF01 and CAF08b, both known to be effective at enhancing immune responses in the neonatal period. CAF01 has been shown to enhance GC reaction, Tfh cells, and ASCs (23) and induce a mixed Th1/Th17 response (24) after immunization of neonatal mice with HA. As for CAF08b, it has been shown that a single immunization with CAF08-adjuvanted respiratory syncytial virus pre-fusion antigen is able to enhance the induction of antigen-specific CD8^+^ T cells and Th1 cells and protect from RSV infection in neonatal mice (26). In fact, CAF08b was specifically designed to enhance Th1 responses in early life through the synergistic engagement of TLR-7/8 and C-type lectin receptor mincle (26). Herein CAF01 and CAF08b were potent adjuvants that enhanced the immune response to both vaccines tested, reaching good dose-sparing effects, although the effects of CAF08b were more pronounced with the HA vaccine. Thus, we observed higher dose sparing effects of CAF08b on Ab response when combined with the influenza HA protein vaccine (40-fold) than with the pneumococcal conjugate vaccine Pn1-CRM_197_ (eightfold). Additionally, CAF01 and CAF08b induced high MN titers, reaching seroprotection (≥20) against influenza, especially CAF08b that reached seroprotection rate in 100% of mice with up to 1/8 of the vaccine dose, and inducing high levels of IgG Abs even with the lowest dose of HA tested, rendering CAF08b as the most promising candidate of the tested adjuvants for dose sparing of HA vaccine in the neonatal mouse model.

We next examined cross-protection against A/Wisconsin/588/2019, a later H1N1pdm09 strain that has undergone antigenic drift relative to A/Michigan/45/2015, potentially impacting existing Ab recognition and neutralization (72, 73). Notably, CAF01 and CAF08b induced high HAI titers, reaching seroprotection (≥40) against this heterologous strain. Specifically, CAF08b, when combined with 2 µg of HA, achieved a 63% cross-reactive seroprotection rate, indicating that the Abs elicited by the 2015 strain with CAF08b effectively inhibited A/Wisconsin/588/2019 hemagglutination. Such cross-reactivity could be attributed to the Abs targeting highly conserved epitopes shared between these two strains (74), or it might be a beneficial outcome of the adjuvant effect, as adjuvants are known to enhance both the magnitude and breadth of the immune response (75, 76). This is consistent with a study in adult mice, where the “IVAX-1” adjuvant combination enhanced the magnitude and breadth of Ab cross-reactivity induced by recombinant influenza hemagglutinin trimer vaccine (77). In addition, when evaluating the persistence of the immune response through ASCs in BM, all fractional doses of HA with CAF01 or CAF08b induced higher numbers of cross-reactive anti-HA IgG^+^ ASCs than the full dose. These adjuvants also increased the number of cross-reactive anti-HA IgG^+^ ASCs in spleen 8 weeks after immunization with HA influenza vaccine. CAF adjuvants are considered depot-forming adjuvants, and the slow release of the vaccine from the depot could promote germinal center reaction for a long period as previously shown with CAF01, where GCs were observed 42 days post-immunization in the groups immunized with CAF01 (78). In our study, we observed a similar effect of these adjuvants only when used with the influenza HA vaccine, but not with the conjugate Pn1-CRM_197_.

Our findings shed light on how the choice of adjuvant interacts with antigen type to shape the immune response, providing critical insights into optimizing vaccine formulations for diverse pathogens. Herein mmCT appeared to be more effective in increasing the protective capacity of Pn1-CRM_197_ than HA. This may be a good fit due to mmCT’s ability to induce Th17 responses (79), which are crucial against pneumococcal colonization (80, 81). In contrast, influenza virus clearance relies more on HA-specific antibodies, but Th1 responses are also essential (82). In this case, CAF08b seems to be appropriate since it has been shown to have Th1-inducing properties (26) as well as enhancing humoral responses. Therefore, different immune responses are required for optimal protection, with some adjuvants being better suited for pneumococcal vaccines and others for influenza vaccines.

It is worth noting that a full dose of either vaccine with the adjuvants elicited significantly higher Ab responses than a full dose of vaccine alone (**Supplementary Figures S9** and **Supplementary Figure S10**), resulting in comparable or higher Ab responses than that elicited by a half-dose of the vaccine with the adjuvants.

It is also important to note that our study evaluated primary Ab responses after a single neonatal immunization. In adult mice, a single dose of Pn1-CRM_197_, whether full or fractional, elicits high, protective Ab levels within 2 weeks. Conversely, in neonatal mice, Pn1-CRM_197_ generated Ab levels comparable to unimmunized controls (**Supplementary Figure S11**). This aligns with human infant data where a multivalent PncCRM_197_ conjugate vaccine, even with aluminum hydroxide adjuvant, induced low Pn-specific Ab responses after the first dose, requiring subsequent immunizations for robust Ab production (83).

It has also been demonstrated that adult mice generate significantly stronger responses to HA influenza vaccine than neonatal mice. Subcutaneous immunization of adult mice with HA alone elicited antibody titers comparable to those seen in neonatal mice immunized with HA adjuvanted with MF59, GLA-SE, IC31, or CAF01. Notably, adjuvantation further enhanced the antibody responses in adult mice (84, 85).

For most vaccines, it is difficult to induce protective Ab levels in neonates or infants with one immunization, even by including adjuvants in the formulation (86). We demonstrated herein that even though Pn1-specific Abs were high, Pn1-specific ASCs in BM remained relatively low, irrespective of the immunizing dose. This is in accordance with our previously published data, where immunization with a pneumococcal conjugate Pnc1-TT induced lower and less persisting polysaccharide-specific ASCs than protein-specific ASCs in the bone marrow (15), suggesting that the responses to conjugate vaccines at an early age seems to be less potent to the polysaccharide moiety of the vaccine than the protein moiety (50). Given the significant increase in vaccine-specific IgG Abs and the improved rate of responders reaching protective levels when the adjuvants used in this study were added to fractional doses of Pn1-CRM_197_ or HA, the results suggest that immunization of neonates twice with fractional doses of Pn1-CRM_197_ or HA and adjuvants could induce highly protective vaccine-specific Ab levels. Lastly, we evaluated the dose-sparing effects of aluminum hydroxide to benchmark our findings with the novel adjuvants since it is the most commonly used adjuvant in pediatric vaccines. We found that there was no difference between the fractional doses of the vaccines with aluminum hydroxide and the full dose of vaccines alone, demonstrating that aluminum hydroxide had no dose-sparing effect with either vaccine, Pn1-CRM_197_ or HA, in our neonatal mouse model. This is in accordance with our previous studies where aluminum hydroxide did not enhance the neonatal immune response after immunization of mice (15, 16), suggesting that aluminum hydroxide is a suboptimal adjuvant for neonates and highlighting the need for adjuvants tailored to this critical age period. It is worth noting that aluminum phosphate is the standard adjuvant used in pediatric pneumococcal conjugate vaccines. Therefore, it is plausible that aluminum phosphate might have elicited stronger or altered immune responses in our model. This consideration is supported by previous studies demonstrating that aluminum hydroxide and aluminum phosphate activate different innate immune pathways (87).

While this study provides important insights into the dose-sparing effects of several novel adjuvants in neonatal vaccination, it has some limitations. First, the experiments were performed in a neonatal mouse model, which, while useful to study early-life immunity, does not fully replicate the human neonatal immune system. However, the immune system of 7-day-old mice corresponds well to those of human neonates, both in terms of T cell function and Ab responses to proteins and pure polysaccharides (49, 50, 88, 89), making the neonatal mouse an important model, especially for *in vivo* studies. Second, we focused on humoral responses, upon which correlates of protection against pneumococcal and influenza infections are based, although recognizing that cellular immune responses likely contribute to protection and vaccine efficacy. We aim to assess cellular responses in future studies. In this study, we follow previously published methodologies and define an MN titer ≥20 as an indicative threshold for seroprotection (58, 59), it is important to note that no absolute MN correlate of protection has been universally defined for all influenza strains or vaccine platforms, and the protective threshold may vary depending on host factors, viral strains, and assay methodology (90). Protective thresholds for pneumococcal bacteremia and lung infection reported in earlier studies were established using both subcutaneous and intranasal immunization, with a different pneumococcal conjugate vaccine (Pnc1-TT) (38, 39, 49). These immunization routes likely elicit both mucosal and systemic antibody responses. In the present study, we have confirmed comparable thresholds in neonatal mice immunized subcutaneously with the vaccine used herein (Pn1-CRM_197_). Given that protection against invasive pneumococcal disease is primarily mediated by serum IgG antibodies (91, 92), the Pn1-specific thresholds associated with protection against bacteremia and lung infection in the mouse model provide a relevant benchmark to evaluate the protective capacity of vaccination formulations and schedules. Importantly, in humans, correlates of protection against pneumococcal disease are based on pneumococcal serotype-specific IgG levels and opsonophagocytic activity (OPA) in serum (93, 94).

Using a neonatal murine model to screen for adjuvants is an effective approach to study the immune response in early life, which can differ significantly from adults. The main advantages of the neonatal mouse model for evaluation of adjuvant effects, vaccine formulations, and vaccination strategies are its sensitivity to detect improved immune responses due to the immaturity of their immune system, access to all key immune compartments, and correlation with the developmental stage of the human neonatal immune system (88, 89). Thus, our model offers a unique target for vaccination and adjuvant studies, and understanding how adjuvants can enhance immune responses in this population is crucial for vaccine development (67). Integrating dose-sparing adjuvants into pediatric vaccines can potentially increase the available supply of vaccine doses. The results presented in this study indicate that mmCT, CAF01, and CAF08b are promising adjuvants in early life vaccination, as a single immunization of neonatal mice with the vaccines Pn1-CRM_197_ or HA with these adjuvants provides antigen dose sparing and enhances the persistence of immune responses. Our results support the potential use of these safe adjuvants in situations when vaccine production capacity is limited, including vaccination of pediatric populations that may be of high risk.

## Data Availability

The raw data supporting the conclusions of this article will be made available by the authors, without undue reservation.

## References

[1] WHO. Vaccines and immunization (2023). Available online at: https://www.who.int/health-topics/vaccines-and-immunizationtab=tab_1 (Accessed September 28, 2023).

[2] WHO. World health statistics Monitoring health for the sdgs, sustainable development goals Vol. 2023 Geneva: World Health Organization (2023). p. 119.

[3] Saso A Kampmann B. Vaccine responses in newborns. Semin immunopathology. (2017) 39:627–42. doi: 10.1007/s00281-017-0654-9, PMID: 29124321 PMC5711983

[4] Mohr E Siegrist C-A. Vaccination in early life: standing up to the challenges. Curr Opin Immunol. (2016) 41:1–8. doi: 10.1016/j.coi.2016.04.004, PMID: 27104290

[5] Pulendran B SAP O’Hagan DT. Emerging concepts in the science of vaccine adjuvants. Nat Rev Drug Discovery. (2021) 20:454–75. doi: 10.1038/s41573-021-00163-y, PMID: 33824489 PMC8023785

[6] Lee W Suresh M. Vaccine adjuvants to engage the cross-presentation pathway. Front Immunol. (2022) 13:940047. doi: 10.3389/fimmu.2022.940047, PMID: 35979365 PMC9376467

[7] Norton EB Bauer DL Weldon WC Oberste MS Lawson LB Clements JD. The novel adjuvant dmLT promotes dose sparing, mucosal immunity and longevity of antibody responses to the inactivated polio vaccine in a murine model. Vaccine. (2015) 33:1909–15. doi: 10.1016/j.vaccine.2015.02.069, PMID: 25765967

[8] Honda-Okubo Y Baldwin J Petrovsky N. Advax-cpG adjuvant provides antigen dose-sparing and enhanced immunogenicity for inactivated poliomyelitis virus vaccines. Pathogens. (2021) 10. doi: 10.3390/pathogens10050500, PMID: 33919442 PMC8143488

[9] Dietrich J Andreasen LV Andersen P Agger EM. Inducing dose sparing with inactivated polio virus formulated in adjuvant CAF01. PloS One. (2014) 9:e100879. doi: 10.1371/journal.pone.0100879, PMID: 24956110 PMC4067388

[10] Martel CJ Agger EM Poulsen JJ Hammer Jensen T Andresen L Christensen D. CAF01 potentiates immune responses and efficacy of an inactivated influenza vaccine in ferrets. PloS One. (2011) 6:e22891. doi: 10.1371/journal.pone.0022891, PMID: 21850242 PMC3151275

[11] Gordon DL Sajkov D Honda-Okubo Y Wilks SH Aban M Barr IG. Human Phase 1 trial of low-dose inactivated seasonal influenza vaccine formulated with Advax™ delta inulin adjuvant. Vaccine. (2016) 34:3780–6. doi: 10.1016/j.vaccine.2016.05.071, PMID: 27342914 PMC4949042

[12] Gorse GJ Grimes S Buck H Mulla H White P Hill H. A phase 1 dose-sparing, randomized clinical trial of seasonal trivalent inactivated influenza vaccine combined with MAS-1, a novel water-in-oil adjuvant/delivery system. Vaccine. (2022) 40:1271–81. doi: 10.1016/j.vaccine.2022.01.034, PMID: 35125219

[13] Reisinger KS Holmes SJ Pedotti P Arora AK Lattanzi M. A dose-ranging study of MF59(^®^)-adjuvanted and non-adjuvanted A/H1N1 pandemic influenza vaccine in young to middle-aged and older adult populations to assess safety, immunogenicity, and antibody persistence one year after vaccination. Hum Vaccines immunotherapeutics. (2014) 10:2395–407. doi: 10.4161/hv.29393, PMID: 25424947 PMC4896790

[14] Molina Estupiñan JL Aradottir Pind AA Foroutan Pajoohian P Jonsdottir I Bjarnarson SP. The adjuvants dmLT and mmCT enhance humoral immune responses to a pneumococcal conjugate vaccine after both parenteral or mucosal immunization of neonatal mice. Front Immunol. (2022) 13:1078904. doi: 10.3389/fimmu.2022.1078904, PMID: 36741402 PMC9896006

[15] Aradottir Pind AA Dubik M Thorsdottir S Meinke A Harandi AM Holmgren J. Adjuvants enhance the induction of germinal center and antibody secreting cells in spleen and their persistence in bone marrow of neonatal mice. Front Immunol. (2214) 2019:10. doi: 10.3389/fimmu.2019.02214, PMID: 31616417 PMC6775194

[16] Aradottir Pind AA Thorsdottir S Magnusdottir GJ Meinke A Del Giudice G Jonsdottir I. A comparative study of adjuvants effects on neonatal plasma cell survival niche in bone marrow and persistence of humoral immune responses. Front Immunol. (2022) 13:904415. doi: 10.3389/fimmu.2022.904415, PMID: 35990686 PMC9381929

[17] Aradottir Pind AA Molina Estupiñan JL Magnusdottir GJ Del Giudice G Jonsdottir I Bjarnarson SP. LT-K63 enhances B cell activation and survival factors in neonatal mice that translates into long-lived humoral immunity. Front Immunol. (2020) 11:527310. doi: 10.3389/fimmu.2020.527310, PMID: 33193301 PMC7644473

[18] Bjarnarson SP Adarna BC Benonisson H Del Giudice G Jonsdottir I. The adjuvant LT-K63 can restore delayed maturation of follicular dendritic cells and poor persistence of both protein- and polysaccharide-specific antibody-secreting cells in neonatal mice. J Immunol (Baltimore Md: 1950). (2012) 189:1265–73. doi: 10.4049/jimmunol.1200761, PMID: 22753937 PMC3496199

[19] Aradottir Pind AA Dubik M Thorsdottir S Meinke A Harandi AM Holmgren J. Adjuvants enhance the induction of germinal center and antibody secreting cells in spleen and their persistence in bone marrow of neonatal mice. Front Immunol. (2019) 10:2214. doi: 10.3389/fimmu.2019.02214, PMID: 31616417 PMC6775194

[20] Christensen D Agger EM Andreasen LV Kirby D Andersen P Perrie Y. Liposome-based cationic adjuvant formulations (CAF): Past, present, and future. J Liposome Res. (2009) 19:2–11. doi: 10.1080/08982100902726820, PMID: 19515003

[21] Christensen D Foged C Rosenkrands I Lundberg CV Andersen P Agger EM. CAF01 liposomes as a mucosal vaccine adjuvant: *In vitro* and *in vivo* investigations. Int J pharmaceutics. (2010) 390:19–24. doi: 10.1016/j.ijpharm.2009.10.043, PMID: 19879346

[22] Olsen AW Theisen M Christensen D Follmann F Andersen P. Protection against Chlamydia promoted by a subunit vaccine (CTH1) compared with a primary intranasal infection in a mouse genital challenge model. PloS One. (2010) 5:e10768. doi: 10.1371/journal.pone.0010768, PMID: 20505822 PMC2874006

[23] Vono M Eberhardt CS Mohr E Auderset F Christensen D Schmolke M. Overcoming the neonatal limitations of inducing germinal centers through liposome-based adjuvants including C-type lectin agonists trehalose dibehenate or curdlan. Front Immunol. (2018) 9. doi: 10.3389/fimmu.2018.00381, PMID: 29541075 PMC5835515

[24] Vono M Mastelic-Gavillet B Mohr E Östensson M Persson J Olafsdottir TA. C-type lectin receptor agonists elicit functional IL21-expressing Tfh cells and induce primary B cell responses in neonates. Front Immunol. (2023) 14:1155200. doi: 10.3389/fimmu.2023.1155200, PMID: 37063899 PMC10102809

[25] van Haren SD Dowling DJ Foppen W Christensen D Andersen P Reed SG. Age-specific adjuvant synergy: dual TLR7/8 and mincle activation of human newborn dendritic cells enables th1 polarization. J Immunol (Baltimore Md: 1950). (2016) 197:4413–24. doi: 10.4049/jimmunol.1600282, PMID: 27793997 PMC7386828

[26] van Haren SD Pedersen GK Kumar A Ruckwardt TJ Moin S Moore IN. CAF08 adjuvant enables single dose protection against respiratory syncytial virus infection in murine newborns. Nat Commun. (2022) 13:4234. doi: 10.1038/s41467-022-31709-2, PMID: 35918315 PMC9346114

[27] Kool M Fierens K Lambrecht BN. Alum adjuvant: some of the tricks of the oldest adjuvant. J Med Microbiol. (2012) 61:927–34. doi: 10.1099/jmm.0.038943-0, PMID: 22174375

[28] Dowling DJ Levy O. Pediatric vaccine adjuvants: components of the modern vaccinologist’s toolbox. Pediatr Infect Dis J. (2015) 34:1395–8. doi: 10.1097/INF.0000000000000893, PMID: 26353029 PMC4931280

[29] Weiser JN Ferreira DM Paton JC. Streptococcus pneumoniae: transmission, colonization and invasion. Nat Rev Microbiol. (2018) 16:355–67. doi: 10.1038/s41579-018-0001-8, PMID: 29599457 PMC5949087

[30] Akkoyunlu M. State of pneumococcal vaccine immunity. Hum Vaccines immunotherapeutics. (2024) 20:2336358. doi: 10.1080/21645515.2024.2336358, PMID: 38567485 PMC10993918

[31] O'Leary ST Campbell JD Ardura MI Bryant KA Caserta MT Espinosa C. Recommendations for prevention and control of influenza in children, 2024-2025: policy statement. Pediatrics. (2024) 154. doi: 10.1542/peds.2024-068507, PMID: 39183669

[32] World Health Organization. Organisation mondiale de la S. In: Weekly epidemiological record, vol. 97. Geneva, Switzerland : World Health Organization (2022). p. 19.

[33] Sparrow E Wood JG Chadwick C Newall AT Torvaldsen S Moen A. Global production capacity of seasonal and pandemic influenza vaccines in 2019. Vaccine. (2021) 39:512–20. doi: 10.1016/j.vaccine.2020.12.018, PMID: 33341308 PMC7814984

[34] Anderson P. Antibody responses to Haemophilus influenzae type b and diphtheria toxin induced by conjugates of oligosaccharides of the type b capsule with the nontoxic protein CRM197. Infect Immun. (1983) 39:233–8. doi: 10.1128/iai.39.1.233-238.1983, PMID: 6600444 PMC347931

[35] Norton EB Lawson LB Freytag LC Clements JD. Characterization of a mutant escherichia coli heat-labile toxin, LT(R192G/L211A), as a safe and effective oral adjuvant. Clin Vaccine Immunol. (2011) 18:546–51. doi: 10.1128/cvi.00538-10, PMID: 21288994 PMC3122563

[36] Lebens M Terrinoni M Karlsson SL Larena M Gustafsson-Hedberg T Kallgard S. Construction and preclinical evaluation of mmCT, a novel mutant cholera toxin adjuvant that can be efficiently produced in genetically manipulated Vibrio cholerae. Vaccine. (2016) 34:2121–8. doi: 10.1016/j.vaccine.2016.03.002, PMID: 26973069

[37] Davidsen J Rosenkrands I Christensen D Vangala A Kirby D Perrie Y. Characterization of cationic liposomes based on dimethyldioctadecylammonium and synthetic cord factor from M. tuberculosis (trehalose 6,6’-dibehenate)-a novel adjuvant inducing both strong CMI and antibody responses. Biochim Biophys Acta. (2005) 1718:22–31. doi: 10.1016/j.bbamem.2005.10.011, PMID: 16321607

[38] Jakobsen H Saeland E Gizurarson S Schulz D Jónsdóttir I. Intranasal immunization with pneumococcal polysaccharide conjugate vaccines protects mice against invasive pneumococcal infections. Infection immunity. (1999) 67:4128–33. doi: 10.1128/iai.67.8.4128-4133.1999, PMID: 10417183 PMC96716

[39] Jakobsen H Schulz D Pizza M Rappuoli R Jónsdóttir I. Intranasal immunization with pneumococcal polysaccharide conjugate vaccines with nontoxic mutants of Escherichia coli heat-labile enterotoxins as adjuvants protects mice against invasive pneumococcal infections. Infection immunity. (1999) 67:5892–7. doi: 10.1128/IAI.67.11.5892-5897.1999, PMID: 10531245 PMC96971

[40] WHO. WHO global influenza surveillance network: manual for the laboratory diagnosis and virological surveillance of influenza. Geneva, Switzerland: World Health Organization (2011) p. 153.

[41] Stephenson I Wood JM Nicholson KG Zambon MC. Sialic acid receptor specificity on erythrocytes affects detection of antibody to avian influenza haemagglutinin. J Med Virol. (2003) 70:391–8. doi: 10.1002/jmv.10408, PMID: 12767002

[42] Hirst GK. The quantitative determination of influenza virus and antibodies by means of red cell agglutination. J Exp Med. (1942) 75:49–64. doi: 10.1084/jem.75.1.49, PMID: 19871167 PMC2135212

[43] Bjarnarson SP. The Generation of immunological memory in early murine life. In: Pneumococcal conjugate vaccination with novel adjuvants by different immunization routes. Faculty of Medicine, University of Iceland, Reykjavík (2014).

[44] Bjarnarson SP Adarna BC Benonisson H Del Giudice G Jonsdottir I. The adjuvant LT-K63 can restore delayed maturation of follicular dendritic cells and poor persistence of both protein- and polysaccharide-specific antibody-secreting cells in neonatal mice. J Immunol. (2012) 189:1265–73. doi: 10.4049/jimmunol.1200761, PMID: 22753937 PMC3496199

[45] Mastelic Gavillet B Eberhardt CS Auderset F Castellino F Seubert A Tregoning JS. MF59 mediates its B cell adjuvanticity by promoting T follicular helper cells and thus germinal center responses in adult and early life. J Immunol. (2015) 194:4836–45. doi: 10.4049/jimmunol.1402071, PMID: 25870238

[46] Sakala IG Honda-Okubo Y Li L Baldwin J Petrovsky N. A M2 protein-based universal influenza vaccine containing Advax-SM adjuvant provides newborn protection via maternal or neonatal immunization. Vaccine. (2021) 39:5162–72. doi: 10.1016/j.vaccine.2021.07.037, PMID: 34362601

[47] Dowling DJ Barman S Smith AJ Borriello F Chaney D Brightman SE. Development of a TLR7/8 agonist adjuvant formulation to overcome early life hyporesponsiveness to DTaP vaccination. Sci Rep. (2022) 12:16860. doi: 10.1038/s41598-022-20346-w, PMID: 36258023 PMC9579132

[48] Honda-Okubo Y Sakala IG André G Tarbet EB Hurst BL Petrovsky N. An Advax-CpG55.2 adjuvanted recombinant hemagglutinin vaccine provides immunity against H7N9 influenza in adult and neonatal mice. Vaccine. (2023) 41:5592–602. doi: 10.1016/j.vaccine.2023.07.061, PMID: 37532610

[49] Jakobsen H Bjarnarson S Del Giudice G Moreau M Siegrist CA Jonsdottir I. Intranasal immunization with pneumococcal conjugate vaccines with LT-K63, a nontoxic mutant of heat-Labile enterotoxin, as adjuvant rapidly induces protective immunity against lethal pneumococcal infections in neonatal mice. Infection immunity. (2002) 70:1443–52. doi: 10.1128/iai.70.3.1443-1452.2002, PMID: 11854231 PMC127807

[50] Jakobsen H Hannesdottir S Bjarnarson SP Schulz D Trannoy E Siegrist CA. Early life T cell responses to pneumococcal conjugates increase with age and determine the polysaccharide-specific antibody response and protective efficacy. Eur J Immunol. (2006) 36:287–95. doi: 10.1002/eji.200535102, PMID: 16385627

[51] Olafsdottir TA Lingnau K Nagy E Jonsdottir I. IC31, a two-component novel adjuvant mixed with a conjugate vaccine enhances protective immunity against pneumococcal disease in neonatal mice. Scandinavian J Immunol. (2009) 69:194–202. doi: 10.1111/j.1365-3083.2008.02225.x, PMID: 19281531

[52] Richter MY Jakobsen H Birgisdottir A Haeuw JF Power UF Del Giudice G. Immunization of female mice with glycoconjugates protects their offspring against encapsulated bacteria. Infection immunity. (2004) 72:187–95. doi: 10.1128/iai.72.1.187-195.2004, PMID: 14688096 PMC343960

[53] Richter MY Jakobsen H Haeuw JF Power UF Jonsdottir I. Protective levels of polysaccharide-specific maternal antibodies may enhance the immune response elicited by pneumococcal conjugates in neonatal and infant mice. Infection immunity. (2005) 73:956–64. doi: 10.1128/iai.73.2.956-964.2005, PMID: 15664938 PMC546934

[54] Jakobsen H Bjarnarson S Giudice GD Moreau M Siegrist C-A Jonsdottir I. Intranasal immunization with pneumococcal conjugate vaccines with LT-K63, a nontoxic mutant of heat-labile enterotoxin, as adjuvant rapidly induces protective immunity against lethal pneumococcal infections in neonatal mice. Infection Immunity. (2002) 70:1443–52. doi: 10.1128/iai.70.3.1443-1452.2002, PMID: 11854231 PMC127807

[55] Aradottir Pind AA Molina Estupiñan JL Magnusdottir GJ Del Giudice G Jonsdottir I Bjarnarson SP. LT-K63 enhances B cell activation and survival factors in neonatal mice that translates into long-lived humoral immunity. Front Immunol. (2020) 11. doi: 10.3389/fimmu.2020.527310, PMID: 33193301 PMC7644473

[56] Bjarnarson SP Jakobsen H Del Giudice G Trannoy E Siegrist CA Jonsdottir I. The advantage of mucosal immunization for polysaccharide-specific memory responses in early life. Eur J Immunol. (2005) 35:1037–45. doi: 10.1002/eji.200425850, PMID: 15756644

[57] Sicca F Martinuzzi D Montomoli E Huckriede A. Comparison of influenza-specific neutralizing antibody titers determined using different assay readouts and hemagglutination inhibition titers: good correlation but poor agreement. Vaccine. (2020) 38:2527–41. doi: 10.1016/j.vaccine.2020.01.088, PMID: 32044163

[58] Ehrlich HJ Müller M Oh HM Tambyah PA Joukhadar C Montomoli E. A clinical trial of a whole-virus H5N1 vaccine derived from cell culture. New Engl J Med. (2008) 358:2573–84. doi: 10.1056/nejmoa073121, PMID: 18550874

[59] Olafsdottir TA Alexandersson KF Sveinbjornsson G Lapini G Palladino L Montomoli E. Age and influenza-specific pre-vaccination antibodies strongly affect influenza vaccine responses in the Icelandic population whereas disease and medication have small effects. Front Immunol. (1872) 2018:8. doi: 10.3389/fimmu.2017.01872, PMID: 29358933 PMC5766658

[60] Hobson D Curry RL Beare AS Ward-Gardner A. The role of serum haemagglutination-inhibiting antibody in protection against challenge infection with influenza A2 and B viruses. J Hyg (Lond). (1972) 70:767–77. doi: 10.1017/s0022172400022610, PMID: 4509641 PMC2130285

[61] Trombetta CM Perini D Mather S Temperton N Montomoli E. Overview of serological techniques for influenza vaccine evaluation: past, present and future. Vaccines (Basel). (2014) 2:707–34. doi: 10.3390/vaccines2040707, PMID: 26344888 PMC4494249

[62] Waldock J Zheng L Remarque EJ Civet A Hu B Jalloh SL. Assay harmonization and use of biological standards to improve the reproducibility of the hemagglutination inhibition assay: a FLUCOP collaborative study. mSphere. (2021) 6:e0056721. doi: 10.1128/msphere.00567-21, PMID: 34319129 PMC8530177

[63] Plotkin SA. Correlates of protection induced by vaccination. Clin Vaccine immunology: CVI. (2010) 17:1055–65. doi: 10.1128/cvi.00131-10, PMID: 20463105 PMC2897268

[64] Allegra P Céline L Thomas C Nicolas C Gerrit. B. Meeting vaccine formulation challenges in an emergency setting: Towards the development of accessible vaccines. Pharmacol Res. (2023) 189. doi: 10.1016/j.phrs.2023.106699, PMID: 36796463

[65] Lofano G Mallett CP Bertholet S O’Hagan DT. Technological approaches to streamline vaccination schedules, progressing towards single-dose vaccines. NPJ Vaccines. (2020) 5:88. doi: 10.1038/s41541-020-00238-8, PMID: 33024579 PMC7501859

[66] Lemoine CH Nidom RV Ventura R Indrasari S Normalina I Santoso KP. Better pandemic influenza preparedness through adjuvant technology transfer: challenges and lessons learned. Vaccines (Basel). (2021) 9. doi: 10.3390/vaccines9050461, PMID: 34063131 PMC8148163

[67] Sakala IG Eichinger KM Petrovsky N. Neonatal vaccine effectiveness and the role of adjuvants. Expert Rev Clin Immunol. (2019) 15:869–78. doi: 10.1080/1744666x.2019.1642748, PMID: 31293189 PMC6678067

[68] Dowling DJ van Haren SD Scheid A Bergelson I Kim D Mancuso CJ. TLR7/8 adjuvant overcomes newborn hyporesponsiveness to pneumococcal conjugate vaccine at birth. JCI Insight. (2017) 2:e91020. doi: 10.1172/jci.insight.91020, PMID: 28352660 PMC5360187

[69] Qadri F Akhtar M Bhuiyan TR Chowdhury MI Ahmed T Rafique TA. Safety and immunogenicity of the oral, inactivated, enterotoxigenic Escherichia coli vaccine ETVAX in Bangladeshi children and infants: a double-blind, randomised, placebo-controlled phase 1/2 trial. Lancet Infect diseases. (2020) 20:208–19. doi: 10.1016/s1473-3099(19)30571-7, PMID: 31757774 PMC6990395

[70] Akhtar M Chowdhury MI Bhuiyan TR Kaim J Ahmed T Rafique TA. Evaluation of the safety and immunogenicity of the oral inactivated multivalent enterotoxigenic Escherichia coli vaccine ETVAX in Bangladeshi adults in a double-blind, randomized, placebo-controlled Phase I trial using electrochemiluminescence and ELISA assays for immunogenicity analyses. Vaccine. (2019) 37:5645–56. doi: 10.1016/j.vaccine.2018.11.040, PMID: 30473185 PMC6717083

[71] Akhtar M Nizam NN Basher SR Hossain L Akter S Bhuiyan TR. dmLT adjuvant enhances cytokine responses to T cell stimuli, whole cell vaccine antigens and lipopolysaccharide in both adults and infants. Front Immunol. (2021) 12:654872. doi: 10.3389/fimmu.2021.654872, PMID: 34054818 PMC8160295

[72] Fan S Kong H Babujee L Presler R Jester P Burke D. Assessment of the antigenic evolution of a clade 6B.1 human H1N1pdm influenza virus revealed differences between ferret and human convalescent sera. EBioMedicine. (2024) 101:105013. doi: 10.1016/j.ebiom.2024.105013, PMID: 38364702 PMC10879773

[73] Al Shammari BR. Sequence and phylogenetic analysis of influenza virus (H1N1pdm2009) circulating in riyadh, Saudi Arabia. J Pure Appl Microbiol. (2024) 18:2380–90. doi: 10.22207/jpam.18.4.11

[74] Padilla-Quirarte HO Lopez-Guerrero DV Gutierrez-Xicotencatl L Esquivel-Guadarrama F. Protective antibodies against influenza proteins. Front Immunol. (2019) 10:1677. doi: 10.3389/fimmu.2019.01677, PMID: 31379866 PMC6657620

[75] Giudice GD Hilbert AK Bugarini R Minutello A Popova O Toneatto D. An MF59-adjuvanted inactivated influenza vaccine containing A/Panama/1999 (H3N2) induced broader serological protection against heterovariant influenza virus strain A/Fujian/2002 than a subunit and a split influenza vaccine. Vaccine. (2006) 24:3063–5. doi: 10.1016/j.vaccine.2006.01.015, PMID: 16464520

[76] Pulendran B S Arunachalam P O’Hagan DT. Emerging concepts in the science of vaccine adjuvants. Nat Rev Drug Discovery. (2021) 20:454–75. doi: 10.1038/s41573-021-00163-y, PMID: 33824489 PMC8023785

[77] Hernandez-Davies JE Dollinger EP Pone EJ Felgner J Liang L Strohmeier S. Magnitude and breadth of antibody cross-reactivity induced by recombinant influenza hemagglutinin trimer vaccine is enhanced by combination adjuvants. Sci Rep. (2022) 12:9198. doi: 10.1038/s41598-022-12727-y, PMID: 35654904 PMC9163070

[78] Pedersen GK Wørzner K Andersen P Christensen D. Vaccine adjuvants differentially affect kinetics of antibody and germinal center responses. Front Immunol. (2020) 11:579761. doi: 10.3389/fimmu.2020.579761, PMID: 33072125 PMC7538648

[79] Larena M Holmgren J Lebens M Terrinoni M Lundgren A. Cholera toxin, and the related nontoxic adjuvants mmCT and dmLT, promote human Th17 responses via cyclic AMP-protein kinase A and inflammasome-dependent IL-1 signaling. J Immunol (Baltimore Md: 1950). (2015) 194:3829–39. doi: 10.4049/jimmunol.1401633, PMID: 25786687

[80] Malley R Trzcinski K Srivastava A Thompson CM Anderson PW Lipsitch M. CD4+ T cells mediate antibody-independent acquired immunity to pneumococcal colonization. Proc Natl Acad Sci United States America. (2005) 102:4848–53. doi: 10.1073/pnas.0501254102, PMID: 15781870 PMC555733

[81] Trzcinski K Thompson CM Srivastava A Basset A Malley R Lipsitch M. Protection against nasopharyngeal colonization by Streptococcus pneumoniae is mediated by antigen-specific CD4+ T cells. Infection immunity. (2008) 76:2678–84. doi: 10.1128/IAI.00141-08, PMID: 18391006 PMC2423086

[82] Chen X Liu S Goraya MU Maarouf M Huang S Chen JL. Host immune response to influenza A virus infection. Front Immunol. (2018) 9:320. doi: 10.3389/fimmu.2018.00320, PMID: 29556226 PMC5845129

[83] Ahman H Käyhty H Tamminen P Vuorela A Malinoski F Eskola J. Pentavalent pneumococcal oligosaccharide conjugate vaccine PncCRM is well-tolerated and able to induce an antibody response in infants. Pediatr Infect Dis J. (1996) 15:134–9. doi: 10.1097/00006454-199602000-00009, PMID: 8822286

[84] Mastelic Gavillet B Eberhardt CS Auderset F Castellino F Seubert A Tregoning JS. MF59 mediates its B cell adjuvanticity by promoting T follicular helper cells and thus germinal center responses in adult and early life. J Immunol (Baltimore Md: 1950). (2015) 194:4836–45. doi: 10.4049/jimmunol.1402071, PMID: 25870238

[85] Vono M Eberhardt CS Mohr E Auderset F Christensen D Schmolke M. Overcoming the neonatal limitations of inducing germinal centers through liposome-based adjuvants including C-type lectin agonists trehalose dibehenate or curdlan. Front Immunol. (2018) 9:381. doi: 10.3389/fimmu.2018.00381, PMID: 29541075 PMC5835515

[86] Levy O Goriely S Kollmann TR. Immune response to vaccine adjuvants during the first year of life. Vaccine. (2013) 31:2500–5. doi: 10.1016/j.vaccine.2012.10.016, PMID: 23085363 PMC4048858

[87] Kooijman S Vrieling H Verhagen L de Ridder J de Haan A van Riet E. Aluminum hydroxide and aluminum phosphate adjuvants elicit A different innate immune response. J Pharm Sci. (2022) 111:982–90. doi: 10.1016/j.xphs.2022.01.014, PMID: 35090866

[88] Siegrist CA. Neonatal and early life vaccinology. Vaccine. (2001) 19:3331–46. doi: 10.1016/s0264-410x(01)00028-7, PMID: 11348697

[89] Siegrist CA Aspinall R. B-cell responses to vaccination at the extremes of age. . Nat Rev Immunol. (2009) 9:185–94. doi: 10.1038/nri2508, PMID: 19240757

[90] Trombetta CM Perini D Mather S Temperton N Montomoli E. Overview of serological techniques for influenza vaccine evaluation: past, present and future. Vaccines. (2014) 2:707–34. doi: 10.3390/vaccines2040707, PMID: 26344888 PMC4494249

[91] Jodar L Butler J Carlone G Dagan R Goldblatt D Kayhty H. Serological criteria for evaluation and licensure of new pneumococcal conjugate vaccine formulations for use in infants. Vaccine. (2003) 21:3265–72. doi: 10.1016/s0264-410x(03)00230-5, PMID: 12804857

[92] Musher DM Chapman AJ Goree A Jonsson S Briles D Baughn RE. Natural and vaccine-related immunity to Streptococcus pneumoniae. J Infect diseases. (1986) 154:245–56. doi: 10.1093/infdis/154.2.245, PMID: 3722865

[93] Organization WH. The immunological basis for immunization series: Module 12. In: Pneumococcal vaccines. WHO, Geneva (2009).

[94] Food US Drug A. Prescribing information: prevnar 20 (Pneumococcal 20-valent conjugate vaccine, diphtheria CRM197 protein). United States: Pfizer Inc. (2023).

