# Supplementary material for: Dose-sparing effects of novel adjuvants and aluminum hydroxide on two different vaccines in a neonatal mouse model
Source: Front Immunol. 2025 Jul 31;16:1646677. doi: 10.3389/fimmu.2025.1646677 (PMC12350264; doi:10.3389/fimmu.2025.1646677)
Supplement: Supplementary file 1 [file DataSheet1.docx]

Supplementary Material

Dose sparing effects of novel adjuvants and alum on two different vaccines in a neonatal mouse model.

**Jenny Lorena Molina Estupiñan^1,2^, Poorya Foroutan Pajoohian^1,2^, Gabriel Kristian Pedersen^3^, Dennis Christensen^3^, Serena Marchi^4^, Emanuele Montomoli^4,5^, Stefanía P. Bjarnarson^1,2^ Ingileif Jonsdottir^1,2^, Audur Anna Aradóttir Pind^1,2^**

^1^Faculty of Medicine, School of Health Sciences, University of Iceland, Reykjavík, Iceland

^2^Department of Immunology, Landspitali, the National University Hospital of Iceland, Reykjavík, Iceland

^3^Statens Serum Institut, Copenhagen, Denmark

^4^Department of Molecular Medicine & Institute for Global Health, University of Siena, Siena, Italy

^5^VisMederi, Siena, Italy.

*** Correspondence:**Audur Anna Aradóttir Pind
audurap@landspitali.is

# Supplementary Figures


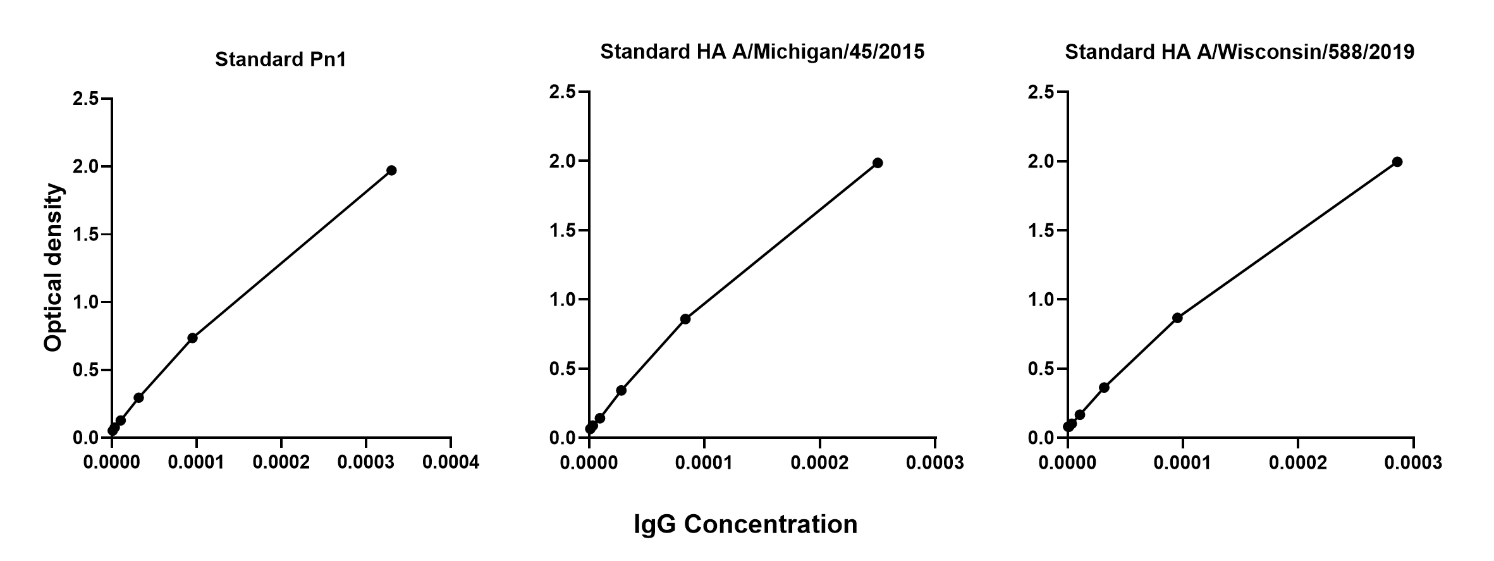


**Supplementary Figure 1. Representative standard curve for ELISA IgG.** Standard curves were made by serial dilutions of reference serum pools from adult mice hyperimmunized with the Pn1-CRM_197_ or influenza HA vaccine to quantify vaccine-specific IgG Abs.

**
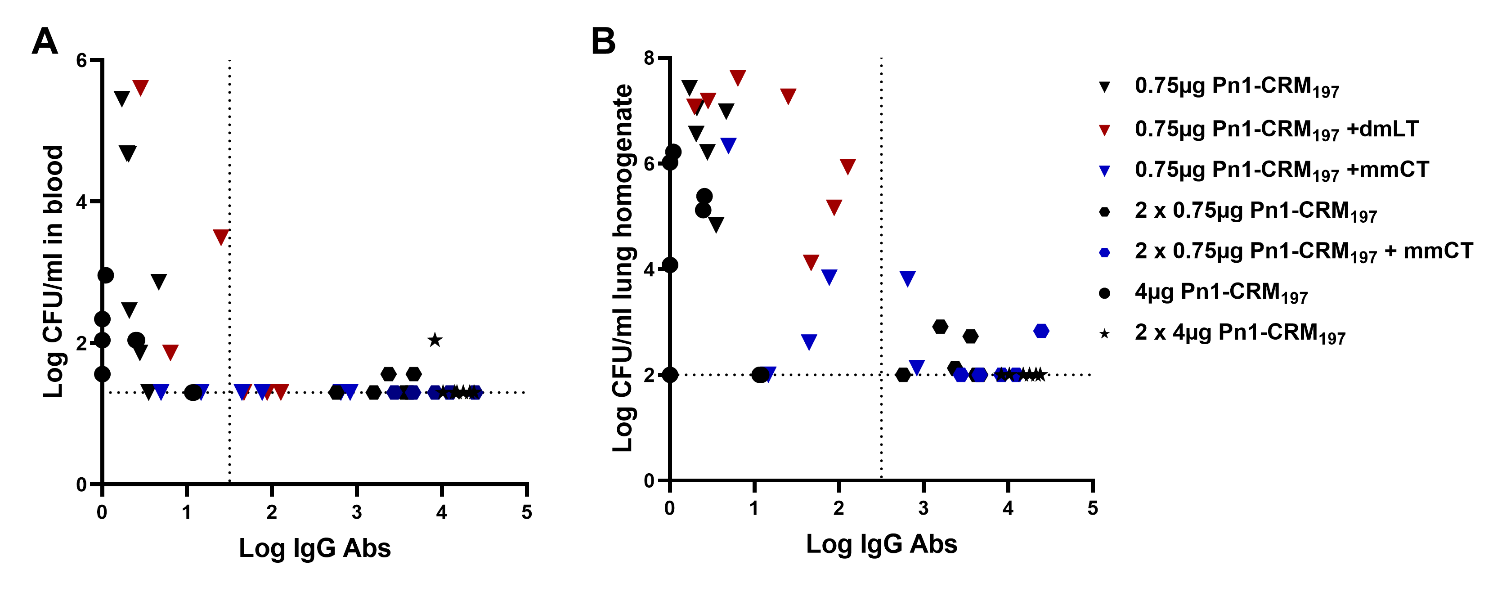
**

**Supplementary Figure 2. Correlation and protective thresholds between pneumococcal bacteremia (A) or lung infection (B) and Pn1-specific IgG Ab titers in serum.** Neonatal mice were immunized once or twice s.c. with 0.75µg or 4µg of the vaccine Pn1-CRM_197_ with or without mmCT and dmLT. The second dose was administered 16 days after the first one, and the infection challenge was performed 2 weeks after the second dose. Pn1-specific IgG Ab (Log EU/mL) in serum were measured 2 days before the challenge. Bacterial density in the blood (A) and the lungs (B) in mice at 24 hours after intranasal challenge with pneumococcal serotype 1 (ATCC 6301). Each symbol represents one mouse. The dotted lines for the Y axis represent the detection limits for CFU/mL. The dotted lines in the X axis represent the protective thresholds Pn1-specific IgG Ab levels, for bacteremia log 1.5 EU/mL (A) and lung infection log 2.5 EU/mL (B).


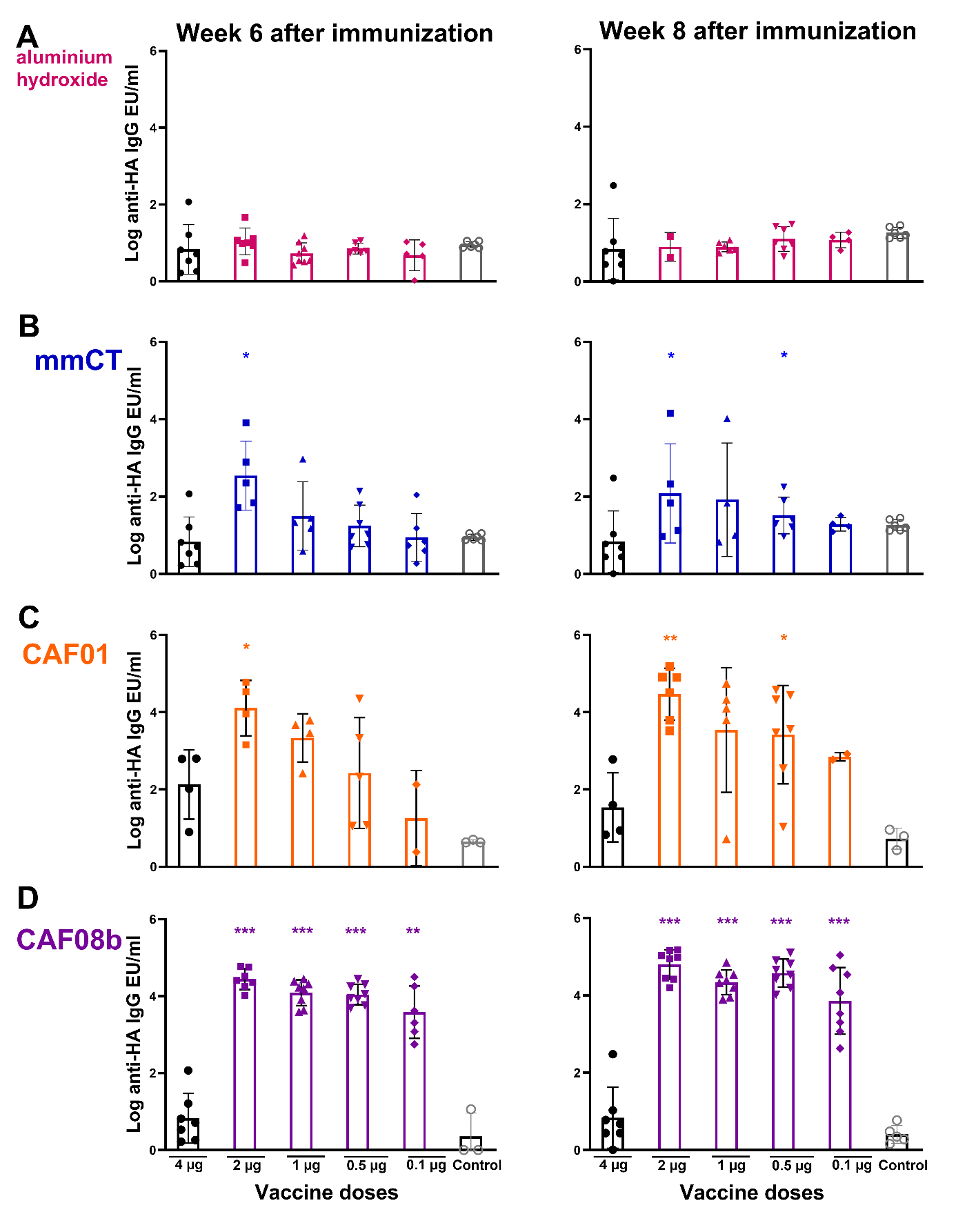


**Supplementary Figure 3. Dose sparing effects of mmCT, CAF01 and CAF08b with HA vaccine.** HA-specific (A/Michigan/45/2015) serum Ab levels 6 and 8 weeks after s.c. immunization of neonatal mice with fractional doses (2µg, 1µg, 0.5µg and 0.1µg) of HA with adjuvants mmCT, CAF01, CAF08b or alum; or with a full dose (4µg) of HA alone. Results are expressed as IgG levels (log mean EU/ml ± SD), and statistical difference was calculated using Mann–Whitney U-test where adjuvant groups were compared to 4µg of vaccine only group. *p ≤ 0.05, **p ≤ 0.01, ***p ≤ 0.001. The results shown are from 3 independent experiments, where mmCT and alum (4 mice/group) were assessed in the same experiment, and CAF01 (4 mice/group) and CAF08b (8 mice/group) in two different experiments.

**
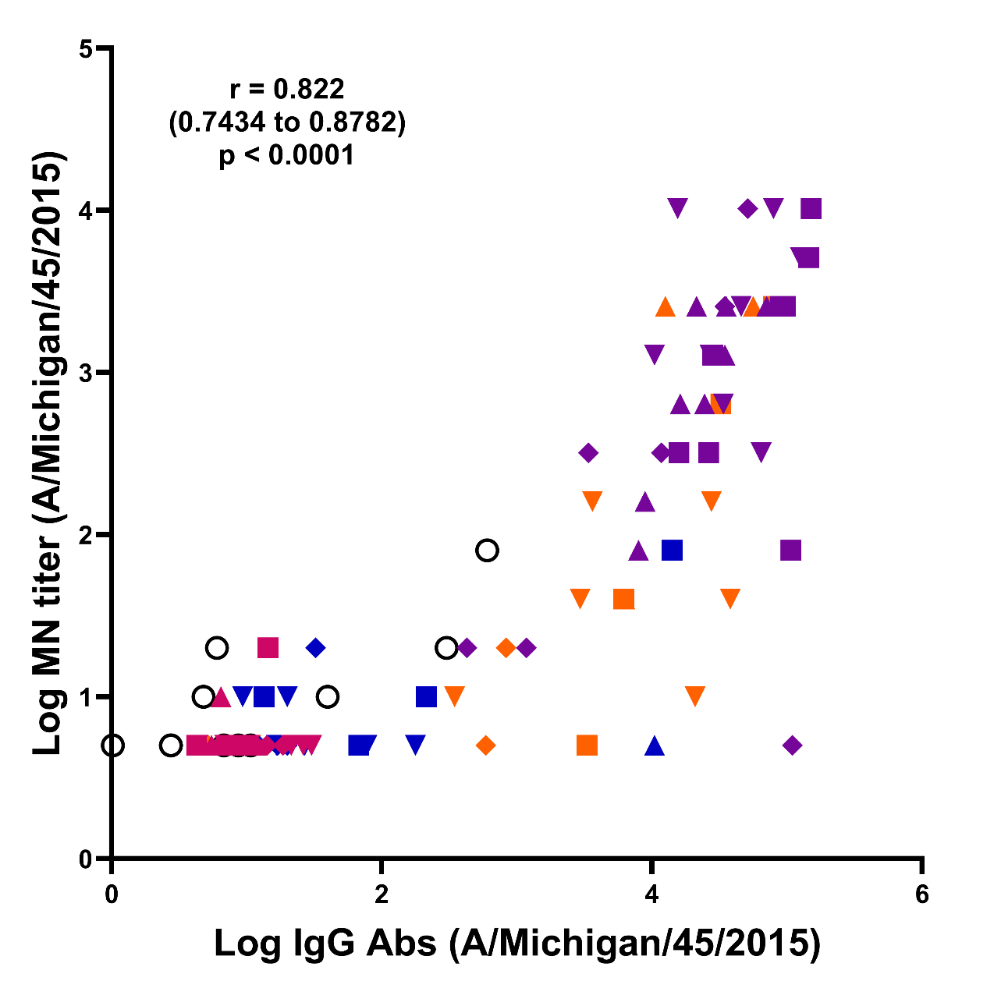
**

**Supplementary Figure 4. Correlation between MN titers – IgG Ab levels.** Spearman correlation was performed between MN titers and ELISA. Open black circles represent full dose HA (4µg), different colors and shapes represent fractional doses of the vaccine with adjuvants as following: aluminum hydroxide in pink, mmCT in blue, CAF01 in orange and CAF08b in purple, 2µg of HA in squares, 1µg of HA in triangles, 0.5µg of HA in inverted triangles, and 0.1µg of HA in diamonds (rhombus).


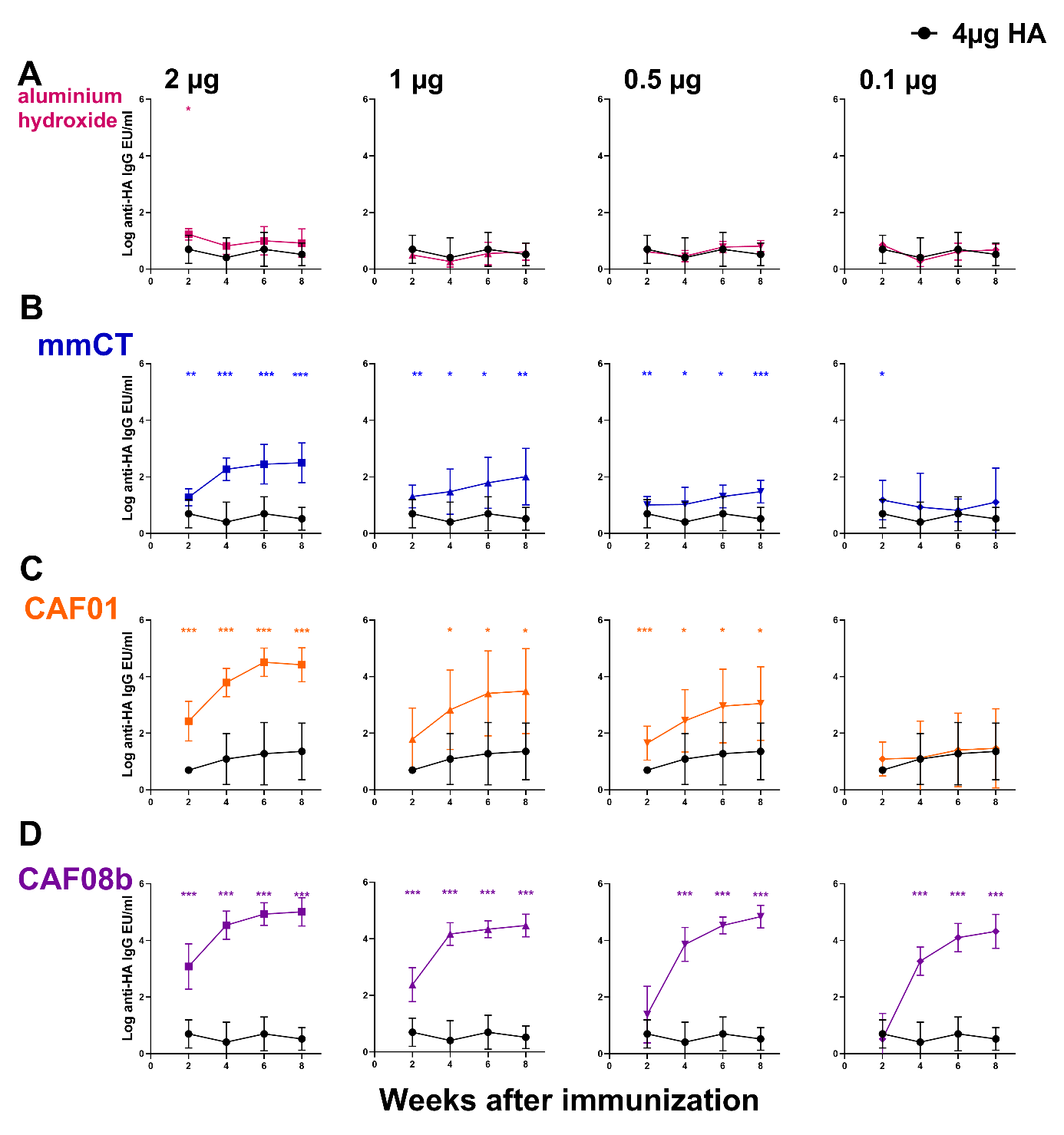


**Supplementary Figure 5. Cross-protective Ab enhancement by mmCT, CAF01 and CAF08b with HA vaccine.** Cross-reactive anti-HA serum Ab levels 2, 4, 6 and 8 weeks after s.c. immunization of neonatal mice with fractional doses (2µg, 1µg, 0.5µg and 0.1µg) of HA with adjuvants aluminium hydroxide (A), mmCT (B), CAF01 (C) or CAF08b (D); or with a full dose (4µg) of HA alone (black filled circle A, B, C and D). Results are expressed as IgG levels (log mean EU/ml ± SD), in 8 mice per group and statistical difference was calculated using Mann–Whitney U-test where adjuvant groups were compared to 4µg of vaccine only group. *p ≤ 0.05, **p ≤ 0.01, ***p ≤ 0.001. The results shown are from 3 independent experiments, where mmCT and aluminium hydroxide were assessed in the same experiment, and the rest of the adjuvants were assessed in different experiments.


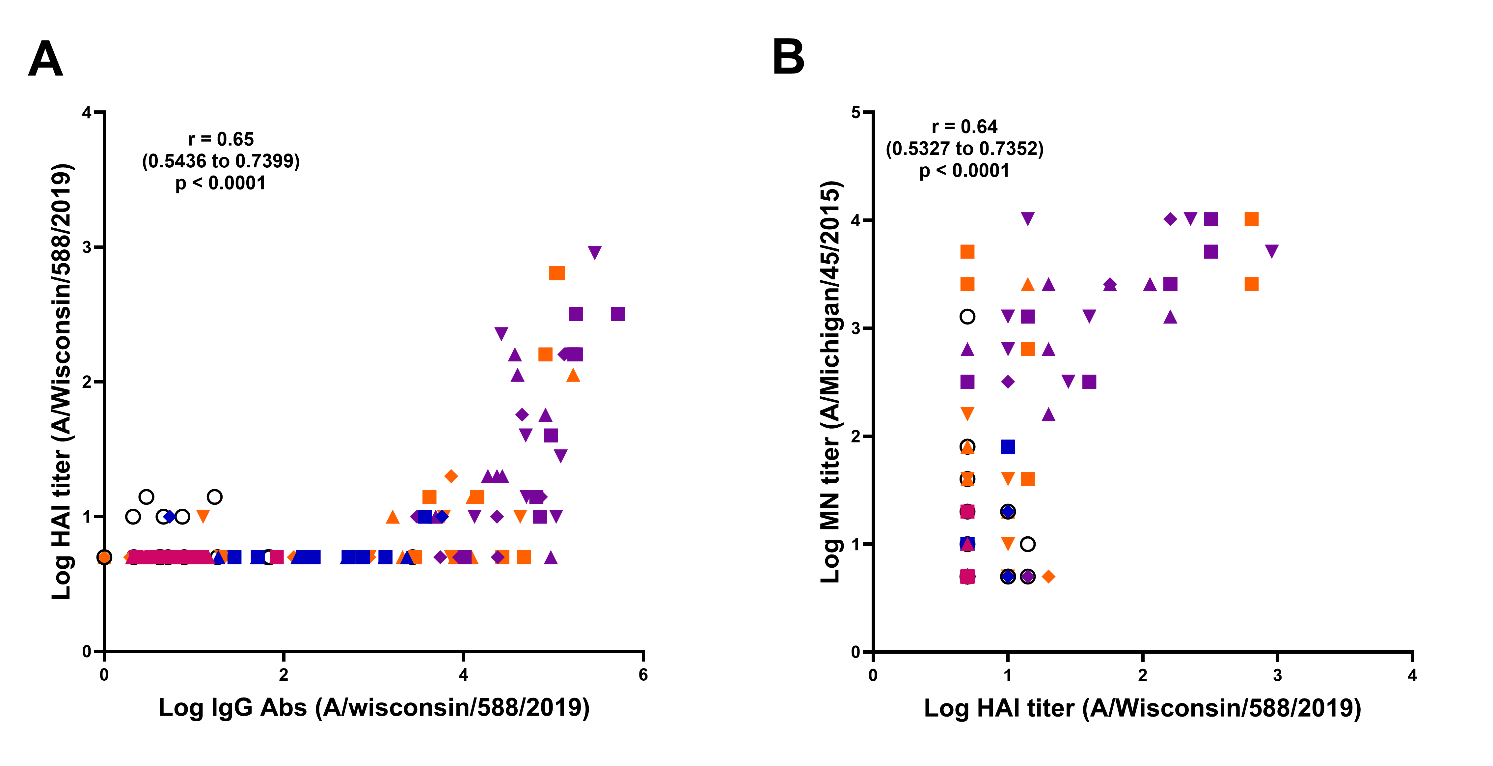


**Supplementary Figure 6. Correlation between HAI titers – IgG Ab titers and MN titers.** Spearman correlation was performed between HAI titers and IgG Ab titers measured with ELISA (A), and MN titers and HAI titers (B). Open black circles represent full dose HA (4µg), different colors and shapes represent fractional doses of the vaccine with adjuvants as following: aluminum hydroxide in pink, mmCT in blue, CAF01 in orange and CAF08b in purple, 2µg of HA in squares, 1µg of HA in triangles, 0.5µg of HA in inverted triangles, and 0.1µg of HA in diamonds (rhombus).

**
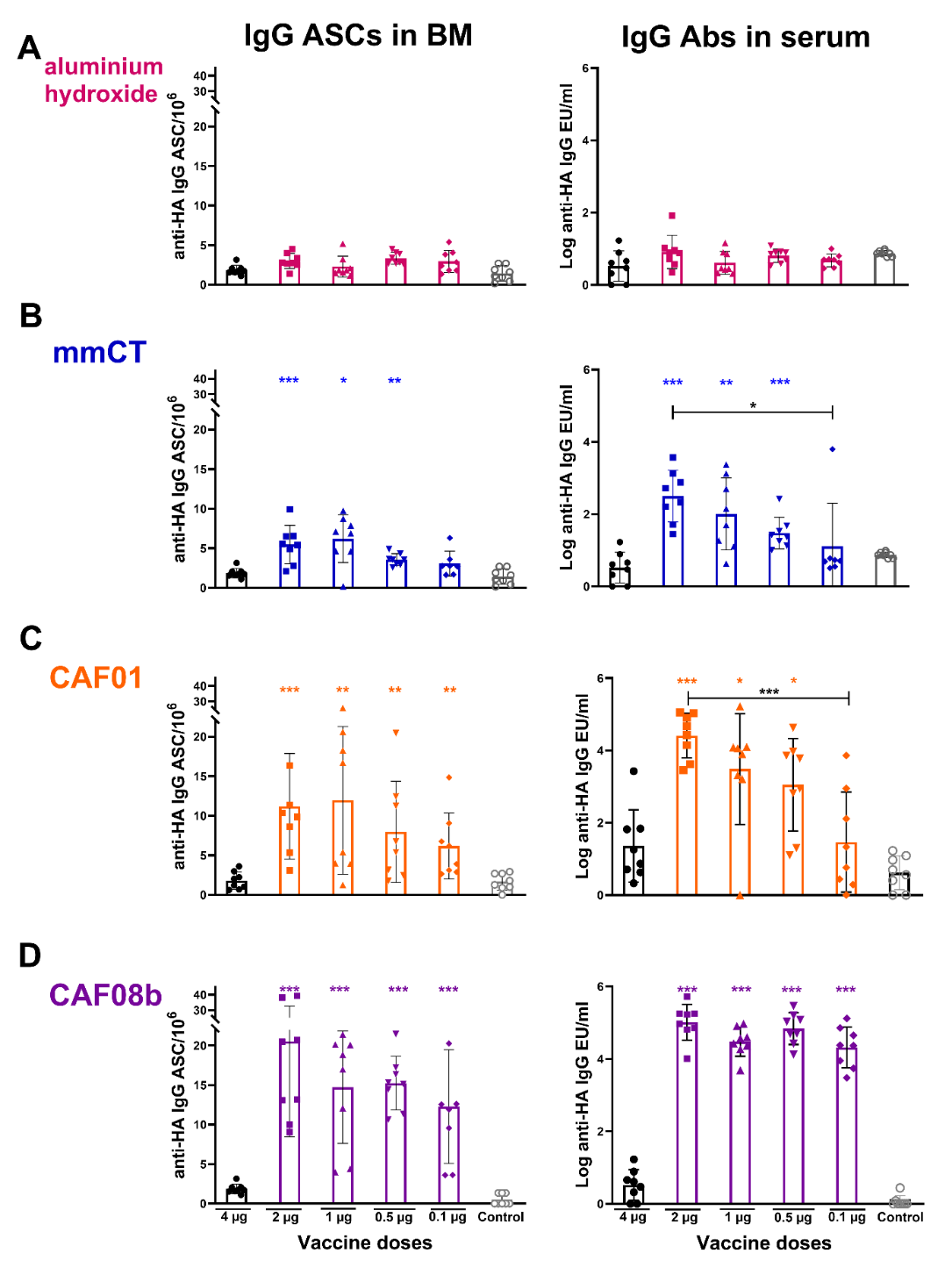
**

**Supplementary Figure 7. Effects of mmCT, CAF01 or CAF08b on persistence of cross-reactive anti-HA IgG^+^ ASCs in bone marrow and IgG Abs in serum.** Number of cross-reactive anti-HA IgG^+^ ASCs (A/Wisconsin/588/2019) in BM and cross-reactive IgG anti-HA (A/Wisconsin/588/2019) levels 8 weeks after s.c. immunization of neonatal mice with fractional doses (2µg, 1µg, 0.5µg and 0.1µg) of HA with adjuvants aluminium hydroxide (A), mmCT (B), CAF01 (C) or CAF08b (D); or with a full dose (4µg) of HA alone (black filled circle A, B, C and D). Results are expressed as number of spots/10^6^ cells (mean ± SD), and IgG levels (log mean EU/ml ± SD), in 8 mice per group and statistical difference was calculated using Mann–Whitney U-test where adjuvant groups were compared to 4µg of vaccine only group (colored stars) and fractional doses of adjuvanted groups were compared between each other (black stars). *p ≤ 0.05, **p ≤ 0.01, ***p ≤ 0.001. The results shown are from 3 independent experiments, where mmCT and aluminium hydroxide were assessed in the same experiment, and the rest of the adjuvants were assessed in different experiments.

**
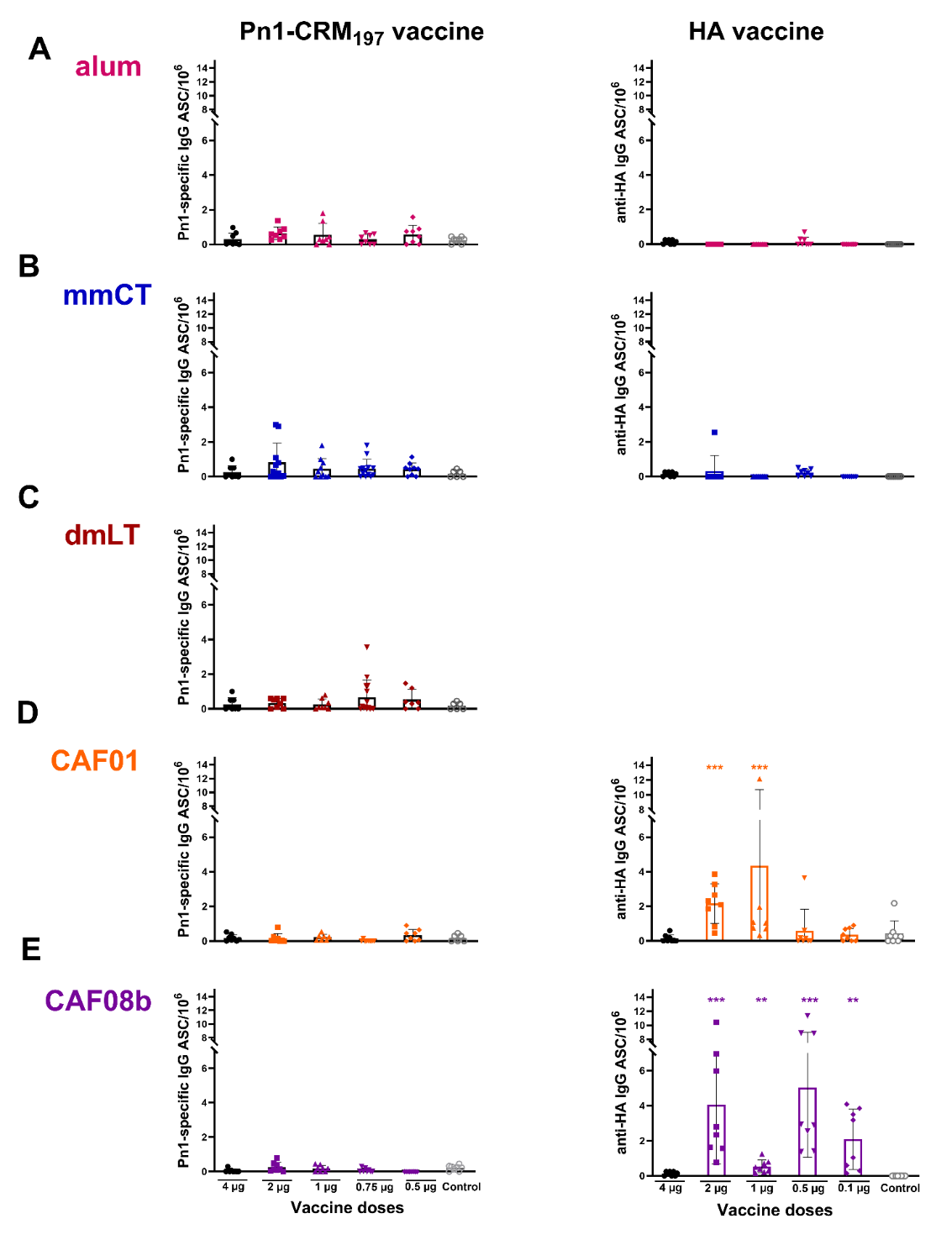
**

**Supplementary Figure 8.** **Effects of mmCT, CAF01 or CAF08b on persistence of Pn1- and HA-specific ASCs in spleen.** Number of Pn1- and HA-specific (A/Wisconsin/588/2019) IgG ASCs in spleen 8 weeks after s.c. immunization of neonatal mice with fractional doses (2µg, 1µg, 0.75, 0.5µg and 0.1µg) of Pn1-CRM_197_ or HA with adjuvants alum (A), mmCT (B), dmLT (C), CAF01 (D) or CAF08b (E), or with a full dose (4µg) of Pn1-CRM_197_ or HA (A/Michigan/45/2015) alone. Results are expressed as number of spots/10^6^ cells (mean ± SD), in 8 mice per group and statistical difference was calculated using Mann–Whitney U-test where adjuvant groups were compared to 4µg of vaccine only group. *p ≤ 0.05, **p ≤ 0.01, ***p ≤ 0.001.


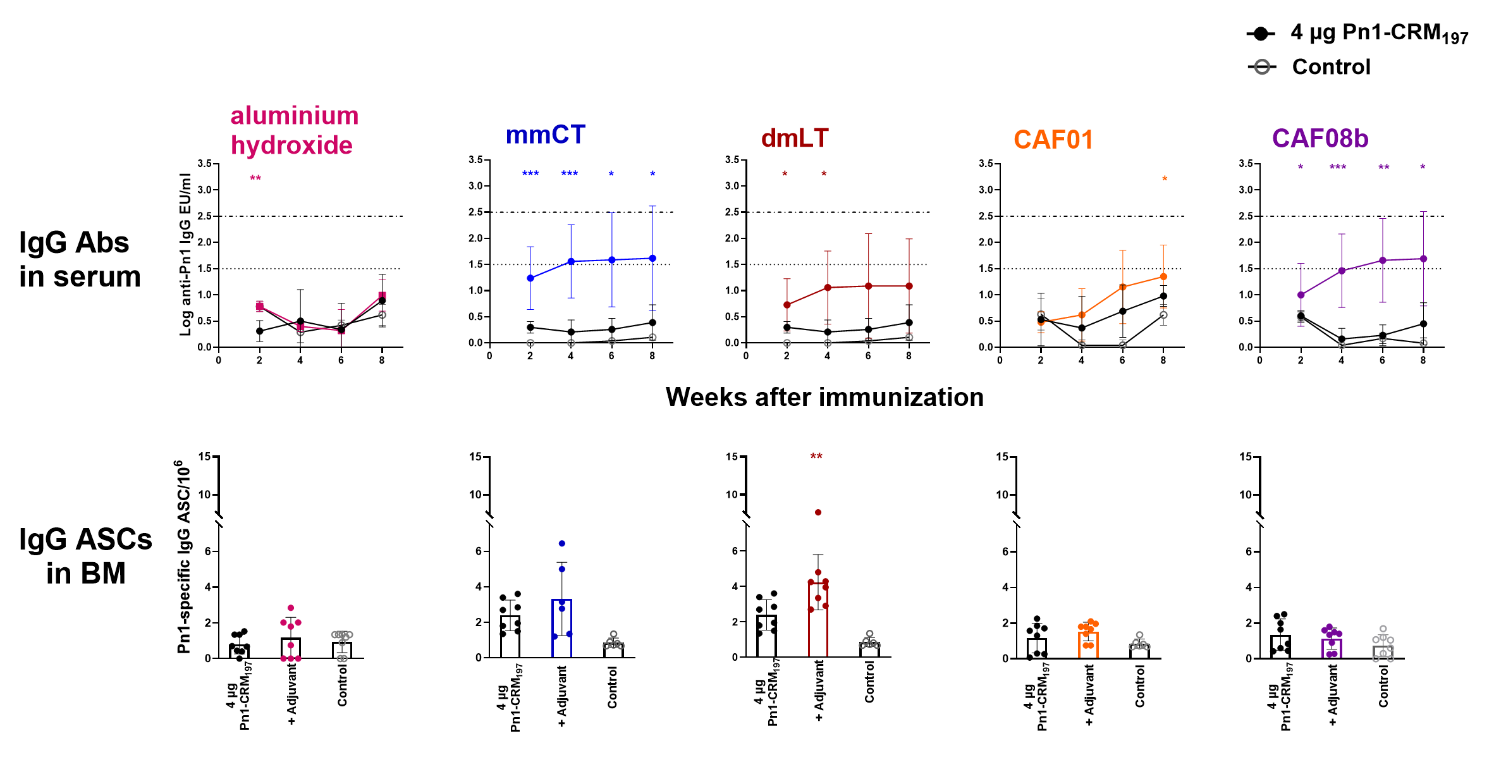


**Supplementary Figure 9. Adjuvants mmCT, dmLT, CAF01 and CAF08b enhance Pn1-specific humoral immune responses following immunization with Pn1-CRM_197_.** Pn1-specific serum Ab levels 2, 4, 6 and 8 weeks after s.c. immunization of neonatal mice with full dose (4µg) of Pn1-CRM_197_ with or without adjuvants aluminium hydroxide (pink), mmCT (blue), dmLT (red), CAF01 (orange) or CAF08b (purple). The full dose (4µg) of Pn1-CRM_197_ alone is represented with black filled circle, and the unimmunized control group with grey open circle. Results are expressed as IgG levels (log mean EU/ml ± SD), in 7-9 mice per group and statistical difference was calculated using Mann–Whitney U-test where adjuvant groups were compared to 4µg of vaccine only group. *p ≤ 0.05, **p ≤ 0.01, ***p ≤ 0.001. The dotted lines represent protective IgG Ab levels for pneumococcal bacteremia (log 1.5) and lung infection (log 2.5). Number of Pn1-specific IgG^+^ ASCs in BM 8 weeks after s.c. immunization is expressed as number of spots/10^6^ cells (mean ± SD).


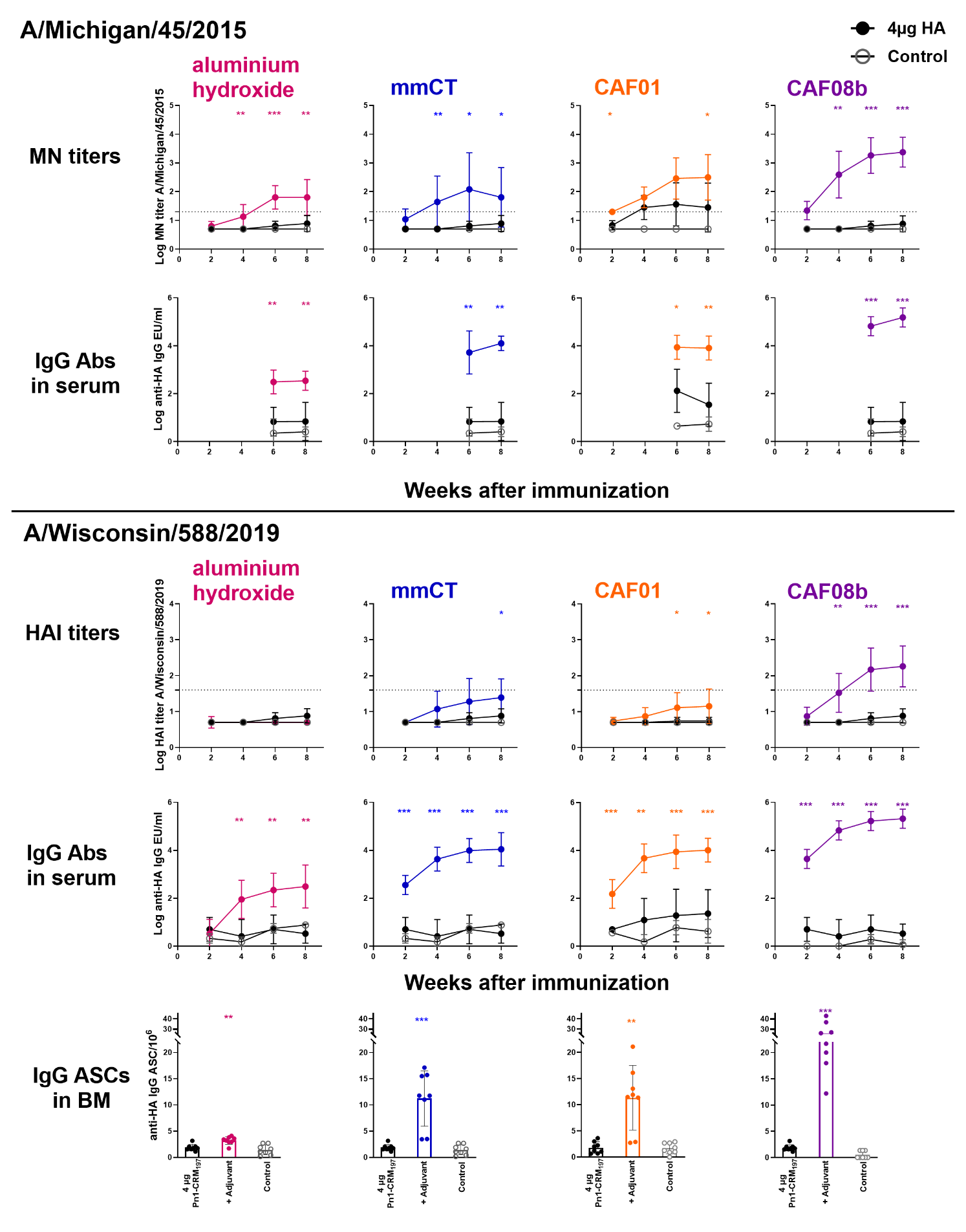


**Supplementary Figure 10. Adjuvants mmCT, CAF01 and CAF08b enhance HA-specific immune responses and cross-reactive responses following immunization with HA.** MN, HAI and IgG Ab assays (ELISA) were performed using serum 2, 4, 6 and 8 weeks after s.c. immunization of neonatal mice with a full dose (4µg) of HA with or without adjuvants aluminium hydroxide (pink), mmCT (blue), CAF01 (orange) or CAF08b (purple). The full dose (4µg) of HA alone is represented with black filled circle and the unimmunized control group with grey open circle. Statistical difference was calculated using Mann–Whitney U-test where adjuvant groups were compared to 4µg of vaccine only group. *p ≤ 0.05, **p ≤ 0.01, ***p ≤ 0.001.

**
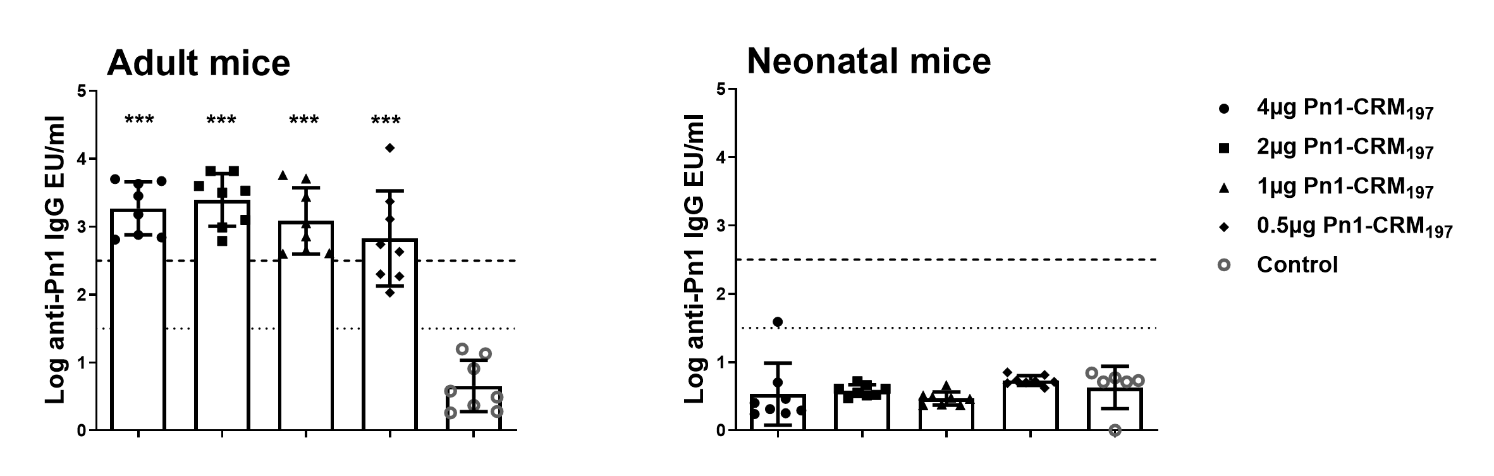
**

**Supplementary Figure 11. IgG Ab levels of adult and neonatal mice with different doses of the vaccine Pn1-CRM_197_ two weeks after immunization.** Adult (5 weeks old) and neonatal mice were immunized with full or fractional doses of the vaccine Pn1-CRM_197_ (4µg, 2µg, 1µg and 0.5µg) and Pn1-specific serum IgG Abs were measured 2 weeks after immunization using ELISA. Results are expressed as IgG levels (log mean EU/ml ± SD), in 7-8 mice per group and statistical difference was calculated using Mann–Whitney U-test where immunized groups were compared to unimmunized control group (*p ≤ 0.05, **p ≤ 0.01, ***p ≤ 0.001). The dotted lines represent protective IgG Ab levels for pneumococcal bacteremia (log 1.5) and lung infection (log 2.5).

# Supplementary Tables

**Supplementary Table 1. Adjuvants evaluated and their effects on immune response in early life.**

| **Adjuvant** | **Constitution** | **Immune response in early life** | **Status** |
| --- | --- | --- | --- |
| mmCT | multiple mutant of Cholera toxin | GCs, ASCs and Abs (1-3) | Preclinical |
| dmLT | double mutant detoxified version of the heat-labile enterotoxin of *Escherichia coli* | GCs, ASCs and Abs (1) | Phase II |
| CAF01 | cationic liposomes DDA with the synthetic immunomodulator TDB | Th1/Th17 (4) | Phase I |
| CAF08b | cationic liposomes DDA with TLR7/8 agonist (3M-052) and TDB | Th1 (5) | Preclinical |
| Alum | aluminum hydroxide | Th2 and Abs (6) | licensed adjuvant |

GC: germinal center, ASC: antibody-secreting cell, Abs: antibodies, DDA: dimethyldioctadecylammonium, TDB: trehalose 6,6’ -dibehenate, TLR: Toll-like receptor.

# References

1. Molina Estupiñan JL, Aradottir Pind AA, Foroutan Pajoohian P, Jonsdottir I, Bjarnarson SP. The adjuvants dmLT and mmCT enhance humoral immune responses to a pneumococcal conjugate vaccine after both parenteral or mucosal immunization of neonatal mice. Front Immunol. 2022;13:1078904.

2. Aradottir Pind AA, Dubik M, Thorsdottir S, Meinke A, Harandi AM, Holmgren J, et al. Adjuvants Enhance the Induction of Germinal Center and Antibody Secreting Cells in Spleen and Their Persistence in Bone Marrow of Neonatal Mice. Frontiers in Immunology. 2019;10(2214).

3. Aradottir Pind AA, Thorsdottir S, Magnusdottir GJ, Meinke A, Del Giudice G, Jonsdottir I, et al. A comparative study of adjuvants effects on neonatal plasma cell survival niche in bone marrow and persistence of humoral immune responses. Front Immunol. 2022;13:904415.

4. Kamath AT, Rochat AF, Christensen D, Agger EM, Andersen P, Lambert PH, et al. A liposome-based mycobacterial vaccine induces potent adult and neonatal multifunctional T cells through the exquisite targeting of dendritic cells. PloS one. 2009;4(6):e5771.

5. van Haren SD, Pedersen GK, Kumar A, Ruckwardt TJ, Moin S, Moore IN, et al. CAF08 adjuvant enables single dose protection against respiratory syncytial virus infection in murine newborns. Nature communications. 2022;13(1):4234.

6. Dowling DJ, Levy O. Pediatric Vaccine Adjuvants: Components of the Modern Vaccinologist's Toolbox. The Pediatric infectious disease journal. 2015;34(12):1395-8.
